# Life History Recorded in the Vagino-cervical Microbiome Along with Multi-omes

**DOI:** 10.1016/j.gpb.2021.01.005

**Published:** 2021-06-09

**Authors:** Zhuye Jie, Chen Chen, Lilan Hao, Fei Li, Liju Song, Xiaowei Zhang, Jie Zhu, Liu Tian, Xin Tong, Kaiye Cai, Zhe Zhang, Yanmei Ju, Xinlei Yu, Ying Li, Hongcheng Zhou, Haorong Lu, Xuemei Qiu, Qiang Li, Yunli Liao, Dongsheng Zhou, Heng Lian, Yong Zuo, Xiaomin Chen, Weiqiao Rao, Yan Ren, Yuan Wang, Jin Zi, Rong Wang, Na Liu, Jinghua Wu, Wei Zhang, Xiao Liu, Yang Zong, Weibin Liu, Liang Xiao, Yong Hou, Xun Xu, Huanming Yang, Jian Wang, Karsten Kristiansen, Huijue Jia

**Affiliations:** 1 BGI-Shenzhen, Shenzhen 518083, China; 2 Shenzhen Key Laboratory of Human Commensal Microorganisms and Health Research, BGI-Shenzhen, Shenzhen 518083, China; 3 Department of Biology, University of Copenhagen, Copenhagen DK-2100, Denmark; 4 Shenzhen Engineering Laboratory of Detection and Intervention of Human Intestinal Microbiome, BGI-Shenzhen, Shenzhen 518083, China; 5 BGI Education Center, University of Chinese Academy of Sciences, Shenzhen 518083, China; 6 China National Genebank, BGI-Shenzhen, Shenzhen 518120, China; 7 BGI-Qingdao, BGI-Shenzhen, Qingdao 266555, China; 8 James D. Watson Institute of Genome Sciences, Hangzhou 310058, China

**Keywords:** Vagino-cervical microbiome, Metagenomic shotgun sequencing, Pregnancy history, Delivery history, Breastfeeding

## Abstract

The vagina contains at least a billion microbial cells, dominated by lactobacilli. Here we perform **metagenomic shotgun sequencing** on cervical and fecal samples from a cohort of 516 Chinese women of reproductive age, as well as cervical, fecal, and salivary samples from a second cohort of 632 women. Factors such as **pregnancyhistory**, **delivery history**, cesarean section, and **breastfeeding** were all more important than menstrual cycle in shaping the microbiome, and such information would be necessary before trying to interpret differences between **vagino-cervical microbiome** data. Greater proportion of *Bifidobacterium breve* was seen with older age at sexual debut. The relative abundance of lactobacilli especially *Lactobacillus crispatus* was negatively associated with pregnancy history. Potential markers for lack of menstrual regularity, heavy flow, dysmenorrhea, and contraceptives were also identified. Lactobacilli were rare during breastfeeding or post-menopause. Other features such as mood fluctuations and facial speckles could potentially be predicted from the vagino-cervical microbiome. Gut and salivary microbiomes, plasma vitamins, metals, amino acids, and hormones showed associations with the vagino-cervical microbiome. Our results offer an unprecedented glimpse into the microbiota of the female reproductive tract and call for international collaborations to better understand its long-term health impact other than in the settings of infection or pre-term birth.

## Introduction

The human body is a supra-organism containing tens of trillions of microbial cells [1]. Studies in human cohorts and animal models have revealed an integral role played by the gut microbiota in metabolic and immunological functions [2]. Disease markers as well as prebiotic or probiotic interventions are being actively developed [3], [4]. The microbiota studies on other mucosal sites such as the vagina and the mouth also have great potential for human health [5], [6], [7]. Despite continued controversy, the presence of microorganisms beyond the cervix (*i.e.*, the upper reproductive tract) is increasingly recognized even in non-infectious conditions [8], [9], [10], [11], [12], with much debated implications for women’s and infants’ health.

Lactobacilli have long been regarded as the keystone species of the vaginal microbiota [13]. Lactic acid produced by these microorganisms helps maintain a low vaginal pH of 3.5–4.5, and wards off pathogenic microorganisms [14]. Prevention of the human immunodeficiency virus (HIV) infection and other sexually transmitted infections (STIs), preterm birth, and bacterial vaginosis (BV) have been major efforts [14], [15], [16]. Germ-free mice treated with *Lactobacillus crispatus* had fewer activated CD4^+^ T cells in the genital tract compared to those treated with *Prevotella bivia*, explaining the difference in HIV acquisition in human cohorts [17]. How vaginal lactobacilli interplay with *Candida albicans* and other related fungi is also an important question for preventing or treating vulvovaginal candidiasis [18]. The fact that human sequences make up more than 90% of the reads in female reproductive tract samples, in contrast to 1% in feces [4], [9], [19], has made metagenomic shotgun sequencing of reproductive tract samples more expensive. To our knowledge, all studies of the vaginal microbiota other than the Human Microbiome Project (HMP) used 16S rRNA gene amplicon sequencing or quantitative polymerase chain reaction (qPCR) [6], [14], [19], [20], which lacked a view of the overall microorganism community including bacteria, archaea, viruses, and fungi [21], as well as the encoded functional capacity. Besides infection, studies on the vaginal microbiota in relation to the current sexual activity or the menstrual cycle have also been reported [13], [22]. However, lasting impacts from other potentially important factors such as sexual debut, pregnancy, and breastfeeding have not been investigated in a large cohort.

As a reservoir of microbes instead of a transient entity [22], the female vagino-cervical microbiota might also reflect conditions in other body sites. It is however not clear whether metabolites and cells in circulation or microbes in other mucosal sites cross-talk with the vagino-cervical microbiome. Intersecting with hormones, metabolic functions, and immune functions, we find it intriguing to explore the potential link of the vagino-cervical microbiome to the brain and the face using questionnaires and measured data that are available for large cohorts.

**Figure 1:**
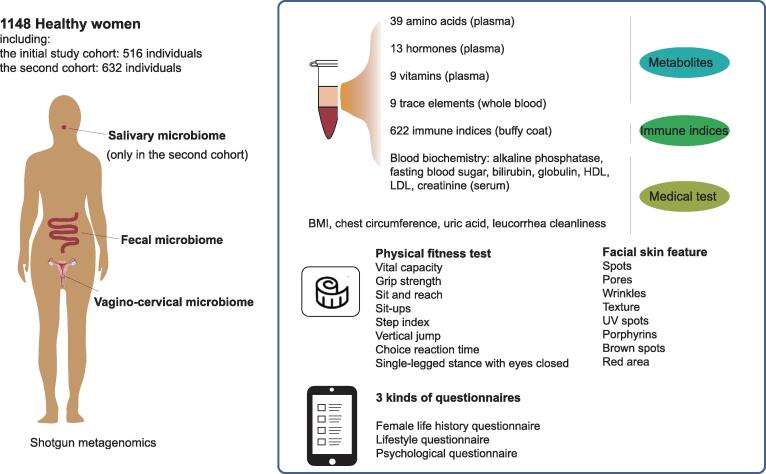
**Schematic overview of the study** The study consists of two cohorts with varied characteristics. The 516 volunteers recruited in 2017 were regarded as the initial study cohort. The 632 volunteers recruited in 2018 were regarded as the second cohort. Vagino-cervical microbiome, fecal microbiome, metabolites, medical test data, physical fitness test data, facial skin features, and three kinds of questionnaires were collected in both cohorts. Salivary microbiome was only available in the second cohort; among medical test results, InBody results, which measure body composition, were only available in the initial study cohort; the woman life history questionnaires differ by a few terms (Table S1). HDL, high density lipoprotein; LDL, low density lipoprotein; BMI, body mass index; UV, ultraviolet.

Here, we report the full spectrum of the vagino-cervical microbiome from 1148 healthy women using metagenomic shotgun sequencing. As part of the 4D-SZ cohort (trans-omics, with more time points in future studies, based in China), we also comprehensively measured the parameters including fecal/salivary microbiomes, plasma metabolites, medical test data, immune indices, physical fitness test, and facial skin imaging, as well as female life history questionnaire, lifestyle questionnaire, and psychological questionnaire (Figure 1). Our work pinpoints other metadata or multi-omes that can predict or be predicted from the microbiota in the female reproductive tract, which would illuminate future designs of population cohorts, mechanistic investigations, and means of intervention.

## Results

### Dominant bacterial and non-bacterial members of the vagino-cervical metagenome

**Figure 2:**
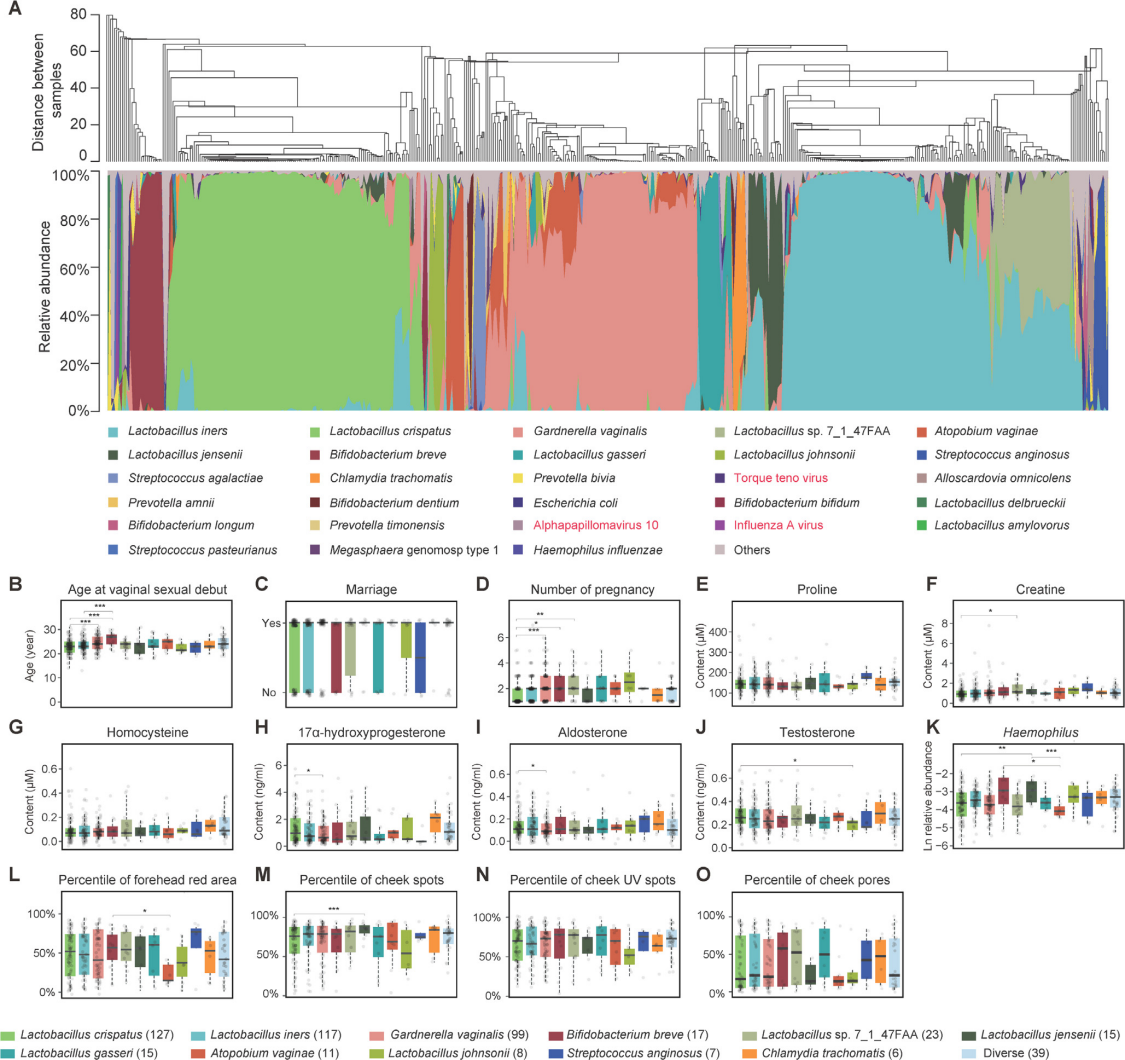
**Vagino-cervical microbiome of the initial study cohort** **A.** The microbial composition in each sample at the species level according to MetaPhlAn2 is shown. The dendrogram in (top) was a result of a centroid linage hierarchical clustering based on Euclidean distances between the microbial composition proportion. Black and red taxa labels denote bacteria and viruses, respectively. **B.**–**O**. Specific multi-omic factors showing significant differences among 12 key vagino-cervical microbiota types estimated by GLM likelihood ratio test (*P* < 0.05, 554 comparisons). Colors of the bars in (B–O) denote the microbiota types as listed at the bottom of the figure. The number of samples per microbiota type is labeled in the parentheses. The horizontal lines with *P* values mark the pairwise post hoc comparisons using Student’s *t*-test (*, *P* < 0.05; **, *P* < 0.01; ***, *P* < 0.001). The genus in (K) belongs to fecal microbiome. Boxes denote the IQR between the first and third quartiles (25th and 75th percentiles, respectively), and the line inside the boxes denotes the median. The whiskers denote the lowest and highest values within 1.5 times the IQR from the first and third quartiles, respectively. GLM, generalized linear model; IQR, interquartile range.

To explore the vagino-cervical microbiome, 516 healthy Chinese women were recruited during a physical examination as the initial study cohort [median age 30, 95% confidence interval (CI): 21–39] (Figure 1; Table S1). Metagenomic shotgun sequencing was performed on the cervical samples, and high-quality non-human reads were used for taxonomic profiling of the vagino-cervical microbiome (Figure 2A; Table S2).

In agreement with the 16S rRNA gene amplicon sequencing data from the USA [13], the vagino-cervical microbiota of this Asian cohort was lactobacilli-dominated. In our study, the species whose relative abundance accounted for 50% or higher in an individual was defined as a community type, while all species accounting for less than 50% of the microbiota in an individual were collectively identified as a diverse community type. The community types characterized by *L. crispatus* and *Lactobacillus iners*, accounting for 24.61% and 22.67% of the individuals, respectively, were the two of the most common types in our initial study cohort (Figure 2A, Figure S1A). *L. iners*, which although traditionally viewed as ‘healthier’ than non-lactobacilli species such as *Gardnerella vaginalis*, has been shown to confer far less protection against bacterial and viral infections than *L. crispatus* [17], [23]. *G. vaginali*s could be detected in 63.57% of the individuals, and in 19.19% of the 516 women, the relative abundance of *G. vaginali*s was equal to or higher than 50% (Figure 2A, Figure S1A; Table S3). *Atopobium vaginae* (recently renamed as *Fannyhessea vaginae* [24]) and *G. vaginalis* are commonly considered to co-occur in BV [20], [25]. This co-occurance was also observed here, *i.e.*, 90.16% of the volunteers with *A. vaginae* in their cervical samples harbored *G. vaginalis* (Figure 2A, Fisher’s exact test, *P* = 1.237E–13, odds ratio = 7.37603). Yet, there were individuals who were dominated by *A. vaginae* (Figure 2A, Figure S1A). Rare subtypes (an identified community type in less than 5% of the 516 individuals) such as those characterized by *Bifidobacterium breve* (3.29%), *Lactobacillus jensenii* (2.91%), *Lactobacillus gasseri* (2.91%), *A. vaginae* (2.13%), *Lactobacillus johnsonii* (1.55%), *Streptococcus anginosus* (1.36%), and *Chlamydia trachomatis* (1.16%) were also detected in this cohort (Figure 2A, Figure S1A). *Streptococcus agalactiae* (Group B *Streptococcus*), a bacterium responsible for neonatal sepsis and recently reported in placenta [10], could be detected in 5.62% of the individuals (Figure 2A; Table S3). Fungal species including *C. albicans*, *Candida glabrata*, *Candida dubliniensis*, and *Candida tropicalis*, commonly believed to result in vulvovaginal candidiasis [26], were also detected in 3.1% of the individuals. Other microorganisms including *P. bivia*, *Escherichia coli*, *Ureaplasma parvum*, human papillomavirus (HPV), herpesviruses, Influenza A virus, and *Haemophilus influenzae* were abundant in some individuals (Figure 2A; Table S3).

Individuals with a vagino-cervical microbiome dominated by different bacteria showed significant differences in some of the questionnaire or omic data [generalized linear model (GLM) likelihood ratio test, overall *P* value < 0.05; Student’s *t*-test between two groups; Figure 2B–O, Figure S1A). The community type characterized by *L. crispatus* was overrepresented in women who had fewer pregnancies than women with the community type *G. vaginalis*, *B. breve*, or *Lactobacillus* sp. 7_1_47FAA (Figure 2D), while individuals of the *B. breve* type had older age at vaginal sexual debut compared to those with the community type *L. crispatus* or *L. iners* (Figure 2B). The plasma concentrations of 17α-hydroxyprogesterone and aldosterone were higher in individuals of the *L. crispatus* type than in the *G. vaginalis* type (Figure 2H and I), while the plasma concentration of testosterone was higher in individuals of the *L. crispatus* type than in the *L. johnsonii* type (Figure 2J). The relative abundance of fecal *Haemophilus* spp. was higher in individuals of the *L. jensenii* type than in the *L. crispatus* and *A. vaginae* community types (Figure 2K). Women of the *B. breve* type had a more serious facial skin problem of red area on the forehead than women with the *A. vaginae* type (Figure 2L), while women with the *L. crispatus* type had fewer spots on the cheeks than women with the *L. jensenii* type (Figure 2M).

According to the metagenomic shotgun data, the mean proportion of non-bacterial sequences was 3.45% (Figure 2A). The vagino-cervical community types of *G. vaginalis*, *L. crispatus*, and *B. breve* showed lower proportions of human sequences than other community types (*e.g.*, *P =* 3.9E–6 between *L. crispatus* and *L. iners* community types, *P =* 1.7E–8 between *G. vaginalis* and *L. iners* community types, and *P* = 0.0036 between *B. breve* and *L. gasseri* community types, Wilcoxon ranked sum test, Figure S1B), suggesting that in future studies lower amounts of sequencing could be used for these compared to other community types, and that species like *L. iners* and *L. gasseri* might be more engaged with human cells.

### Factors associated with the vagino-cervical microbiome

**Figure 3:**
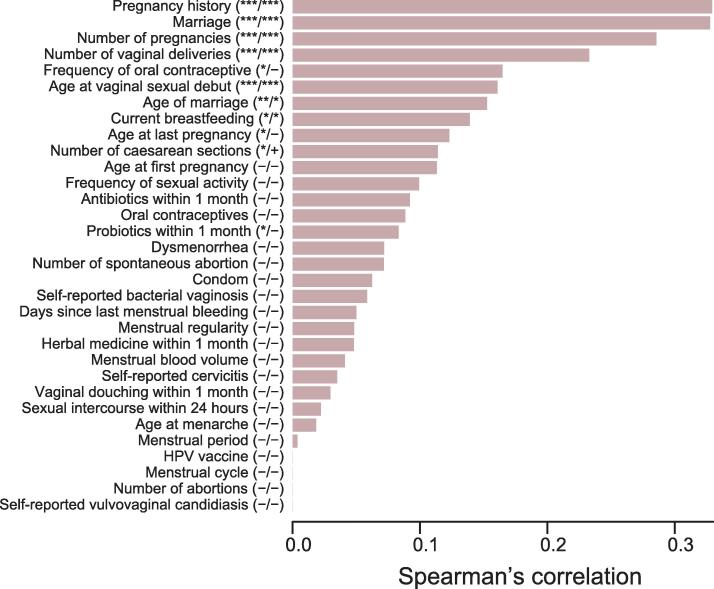
**Factors from the female life history questionnaire influencing the vagino-cervical microbiome in the initial study cohort** Female life history questionnaire entries on the vagino-cervical microbiome, ordered according to their respective 5-fold cross-validated random forest importance on the microbiome composition. X-axis (length of the bar) is the model performance measured as the Spearman’s correlation coefficient between the prediction and measurement. In the parentheses after Y-axis labels, the first column symbols are *P* values with 999 permutations, and the second column symbols are *Q* values for BH method (32 comparisons). “−” denotes value ≥ 0.1, “+” denotes 0.05 ≤ value < 0.1, “*” denotes value < 0.05, “**” denotes value < 0.01, and “***” denotes value < 0.001. BH, Benjamini-Hochberg; HPV, human papillomavirus.

We computed the prediction value (5-fold cross-validated random forest model) of each factor independently in the female life history questionnaire on the relative abundance of vagino-cervical microbiome data, and found the most important factors to be pregnancy history, marriage, number of pregnancies, number of vaginal deliveries, and age at vaginal sexual debut, followed by age of marriage, and current breastfeeding [*P* < 0.05 with 999 permutations and *Q* < 0.05 for Benjamini-Hochberg (BH) method] (Figure 3, Figure S2). None of these factors significantly correlated with one another (Figure S3; Table S4).

These important factors for the vagino-cervical microbiome were validated in an independent cohort of 632 individuals that differed in age distribution as well as sequencing mode (median age 32, 95% CI: 18–47; paired-end 100 bp instead of single-end 50 bp for vagino-cervical samples) (Figure 1, Figure S3; Tables S1–S3). Questionnaire entries such as pregnancy history, marital status, current breastfeeding, and mode of the most recent delivery (caesarean or vaginal) were again found as the most important factors to exhibit correlations with the vagino-cervical microbiome (Figure S3). Duration of current breastfeeding emerged as a strong predictor of the vagino-cervical microbiome composition, which reflected the differences in questionnaire design that was only available in the second but not the first cohort (Figures S3 and S4; Table S1). The prediction value of menstrual cycle was slightly augmented with the presence of postmenopausal women in the second cohort (Figure 3, Figure S3).

Forty-five of the volunteers in the second cohort were postmenopausal (median age 54, 95% CI: 44–64), an age group untouched by HMP. Metagenomic shotgun data revealed diminished lactobacilli in their vagino-cervical microbiome, while the mean proportion of viral sequences reached 37% (Figure S5). HPV was not the most abundant or prevalent virus in these postmenopausal individuals; herpes simplex virus (HSV), phages, and torque teno virus (TTV) were also detected (Figure S5). The endogenous retrovirus was likely part of the human genome, which would need further validation (Figure S5). In contrast, there was no sign of more *C. albicans* or other fungi, suggesting that the lack of glycogen in postmenopausal individuals might also have counteracted fungal growth (Figure S5). Three of the individuals were dominated by *L. crispatus* and two of the individuals by *L. iners*, none of whom was reported to receive hormone replacement therapy, implying genuine individual differences (Figure S5C). The non-lactobacilli bacterial species are also known as vaginal or salivary species, with overgrowth of *E. coli* in two of the individuals (Figure S5C). The taxonomic profile remained robust when we arbitrarily trimmed the paired-end 100 bp data to single-end 50 bp or single-end 100 bp (Spearman’s correlation coefficient = 0.998 between Pseudo-SE50 and Pseudo-SE100, 0.993 between Pseudo-SE50 and PE100) (Figure S6).

**Figure 4:**
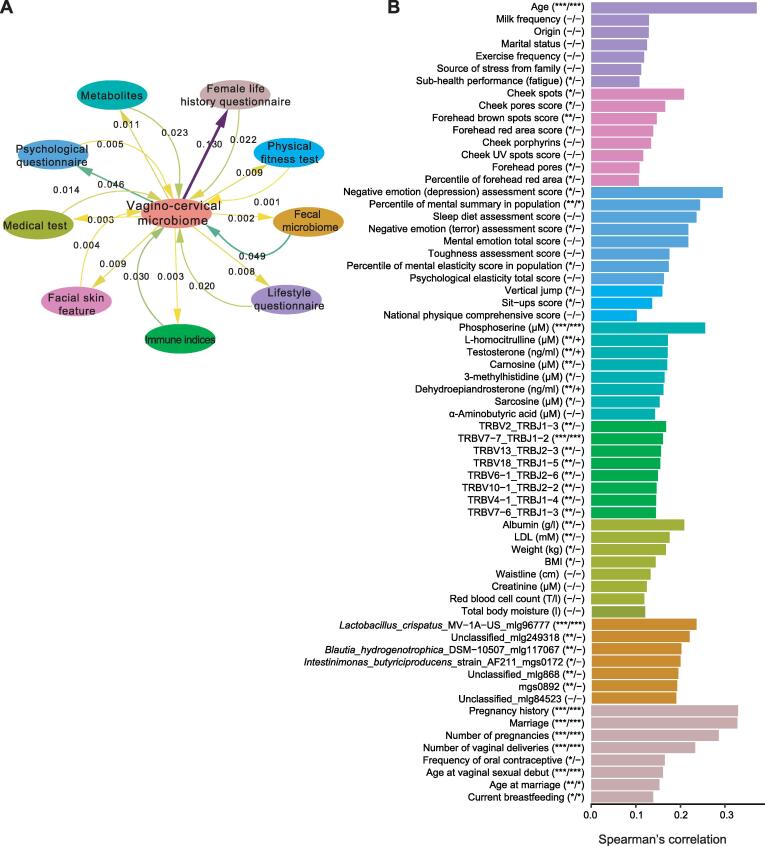
**Global view of factors influencing the vagino-cervical microbiome in the initial study cohort** **A.** Predicting the vagino-cervical microbiome from each omic dataset and *vice versa* using stepwise redundancy analysis. Numbers on the straight arrows indicate adjusted R-squared from vagino-cervical microbiome to multi-omic data; numbers on the curved arrows indicate adjusted R-squared from multi-omic data to vagino-cervical microbiome. **B.** Top 8 factors in each type of multi-omic data that are predicted by the vagino-cervical microbiome. Each covariate from the multi-omic data is detailed in Table S1. X-axis (length of the bar) is the model performance measured as the Spearman’s correlation coefficient between the prediction and measurement.  In the parentheses after Y-axis labels, the first column symbols are *P* values with 999 permutations, and the second column symbols are *Q* values for BH method (32 comparisons). “−” denotes value ≥ 0.1, “+” denotes 0.05 ≤ value < 0.1, “*” denotes value < 0.05, “**” denotes value < 0.01, and “***” denotes value < 0.001.

As a whole, the vagino-cervical microbial composition showed the greatest explained variances for these questionnaire data collected for the vagino-cervical samples, followed by other data collected on the same day, such as fecal microbiome composition, psychological questionnaire, plasma metabolites, immune indices, facial skin imaging, medical test data, and physical fitness test results (Figure 4A). Age, pregnancy history, marriage, and number of pregnancies were the most significant factors among all the multi-omic data [with the largest Spearman’s correlation coefficients between random forest cross validation (RFCV) prediction and observed data, *P* < 0.001, and *Q* < 0.001; Figure 4B]. *L. crispatus* in the fecal microbiome was most predictive of the vagino-cervical microbiome (*P* < 0.001 and *Q* < 0.001); plasma phosphoserine (*P* < 0.001 and *Q* < 0.001), L-homocitrulline (*P* < 0.01 and 0.05 ≤ *Q* < 0.1), and testosterone (*P* < 0.01 and 0.05 ≤ *Q* < 0.1) were ranked at the top among metabolites (Figure 4B); weaker signals were observed for serum albumin and low density lipoprotein (LDL) in medical test data (*P* < 0.01 but *Q* ≥ 0.1) and spots on the cheeks in facial skin imaging (*P* < 0.05 but *Q* ≥ 0.1) (Figure 4B).

### Specific influences from pregnancy history, contraception, and menstrual symptoms

**Figure 5:**
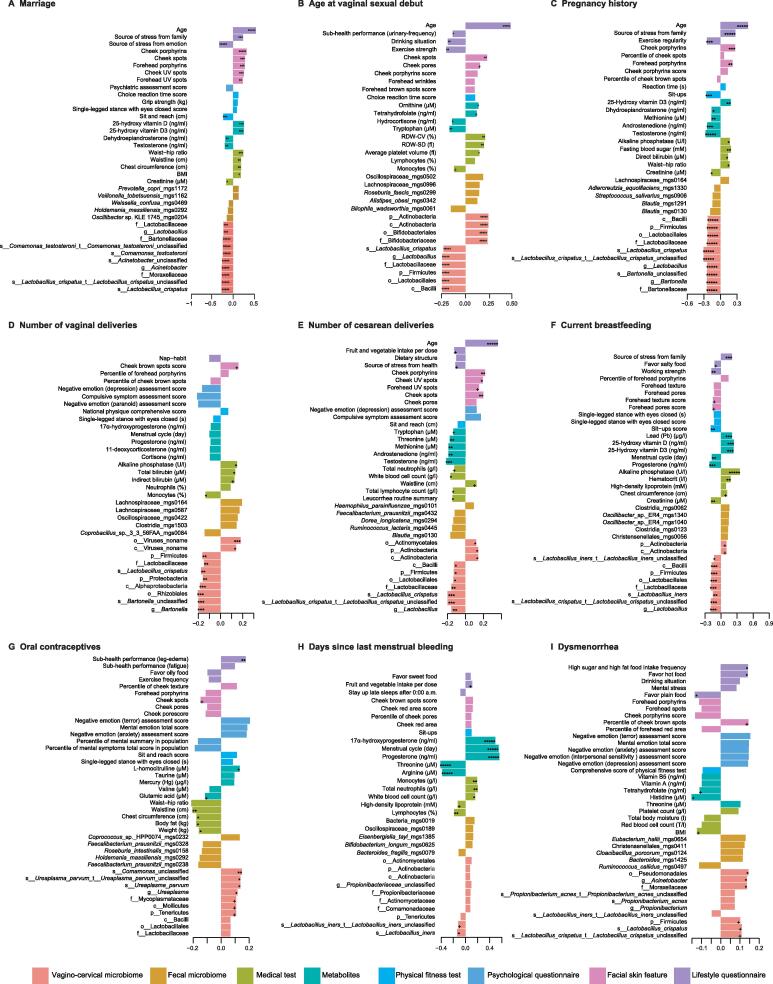
**Specific influencesofreproductive factors on multi-omic data in the initial study cohort** The factors shown are marriage (**A**), age at vaginal sexual debut (**B**), pregnancy history (**C**), number of vaginal deliveries (**D**), number of cesarean deliveries (**E**), current breastfeeding (**F**), oral contraceptives (**G**), days since last menstrual bleeding (**H**), and dysmenorrhea (**I**). The bars are colored according to the type of omic data, as shown in Figure 3. The metabolites such as amino acids, hormones, and vitamins were measured in plasma, and trace elements were measured in whole blood. The blood biochemistry such as alkaline phosphatase, fasting blood sugar, direct bilirubin, creatinine, total bilirubin, and HDL was measured in serum. Each covariate from the multi-omic data is detailed in Table S1. The length of the bars represents the Spearman’s correlation coefficient between the respective factor and the multi-omic data. Average rank selects 13,425 edges from 66,301 associations, including 523 edges for vagino-cervical microbiome, 11,775 for fecal microbiome, 321 for metabolites, 280 for medical test, 70 for physical fitness test, 137 for facial skin feature, and 319 for lifestyle questionnaire. +, 0.05 ≤ *Q* < 0.1; *, *Q* < 0.05; **, *Q* < 0.01; ***, *Q* < 0.001; ****, *Q* < 0.0001; *****, *Q* < 0.00001.

Marital status was one of the most significant factors to associate with the vagino-cervical microbiome (Figure 3, Figure S3). The married individuals showed negative correlations with relative abundances of *L. crispatus*, *Comamonas testosteroni*, and *Acinetobacter* spp. (Spearman’s correlation, *Q* < 0.01) (Figure 5A; Table S6). Compared to unmarried women, married women had higher concentrations of plasma 25-hydroxy vitamin D, and their plasma testosterone, dehydroepiandrosterone (DHEA), and creatinine were lower (Figure 5A; Table S6). *C. testosteroni* TA441, which at the species level was correlated with being married, has been reported to degrade testosterone [27]. The age at vaginal sexual debut showed positive correlations with Bifidobacteriaceae (which includes *G. vaginalis* and *B. breve*) (Figure 5B; Table S4), and negative associations with *L. crispatus* (Figure 5B; Tables S5 and S6), consistent with analyses based on dominant bacteria (Figure 2B).

Similarly, the women who went through pregnancy showed relatively less *Lactobacillus*, especially *L. crispatus* compared to nullipara, as well as less *Bartonella* in the vagino-cervical microbiome (Figure 5C; Table S6). We observed increased concentrations of plasma vitamin D3 in women with previous pregnancy, but lower concentrations of plasma testosterone, androstenedione, dehydroepiandrosterone, and methionine (Figure 5C). Vaginal deliveries were associated with decreased *L. crispatus* and *Bartonella* species (Figure 5D; Table S6); cesarean section deliveries were also correlated with less *L. crispatus*, but higher abundance of Actinobacteria (Figure 5E; Table S6). In addition, cesarean sections were correlated with lower plasma concentrations of testosterone, androstenedione, methionine, threonine, and tryptophan, as well as a larger waistline, lower neutrophil and lymphocyte counts, and abnormal leucorrhoea (Figure 5E). Caesarean section deliveries were also associated with lower facial skin score ranking such as ultraviolet (UV) spots and porphyrins, while those with vaginal deliveries appeared with less brown spots on the cheeks (Figure 5D and E).

A total of 137 volunteers (45.99% from the initial cohort, 54.01% from the second cohort) happened to be actively breastfeeding (Figure 5F, Figure S4). Those who were within the first two years of delivery often lacked lactobacilli, especially *L. crispatus*, and were dominated by viral sequences or BV-related species such as *Prevotella* spp. and *Atopobium* spp. (Figure S4). Only 6.57% of the individuals harbored > 50% *L. crispatus*, which would represent > 20% of the cohort for non-pregnant, non-breastfeeding Asia women of reproductive age (Figures 2 and 5F). In addition, 23.36% of the breastfeeding individuals were dominated by *L. iners*, and 18.25% individuals who had > 50% *G. vaginalis*, similar to the overall distribution (Figure 2, Figure S4). Alkaline phosphatase, plasma vitamin D, and lead (Pb) were found to be higher in the breastfeeding individuals, combined with slight reduction of progesterone and lack of menses in lactating women (Figure 5F).

**Figure 6:**
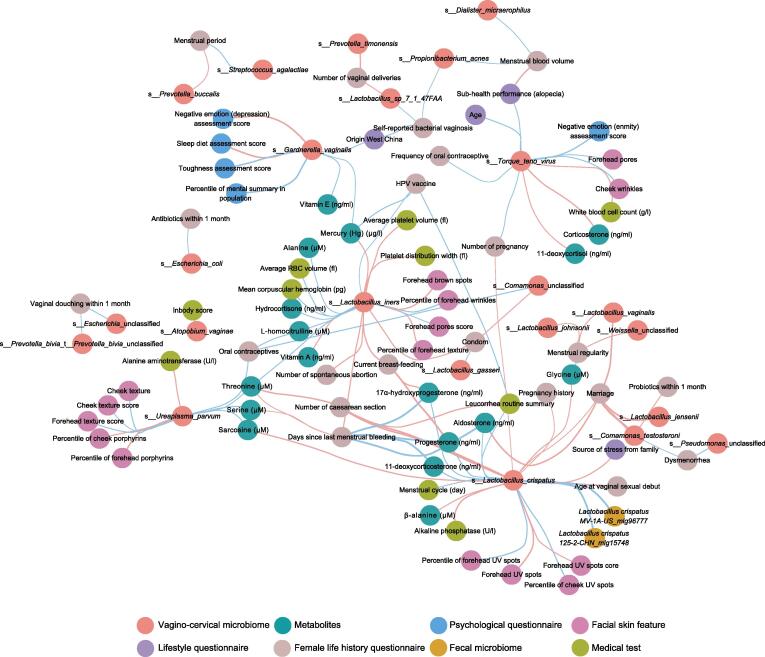
**Wisdom of the crowds for the association network between vagino-cervical microbial species and multi-omic data in the initial study cohort** Results from GLM with penalty (cv.glmnet), random forest cross validation, and Spearman’s correlation are integrated and then visualized in Cytoscape. Red edges indicate negative associations; cyan edges indicate positive associations.

We found multiple significant associations between the contraceptive methods of participants and their vagino-cervical microbiome. Condom usage showed negative correlations with *L. iners* and *Comamonas* species, and positive associations with *L. gasseri* (Figure 6; Tables S5 and S6). Oral contraceptives, although still rare in our cohort, were associated with increased abundances of *L. iners*, *U. parvum*, and *Comamonas* species (Figures 5G and 6; Tables S5 and S6). *U. parvum* is a bacterium commonly isolated from pregnant women [28] and recently reported in the lower respiratory tract of preterm infants [29] and in preterm placenta [10]. *Comamonas* has been implicated in the fecundity in *Caenorhabditis elegans* and identified as a marker for infertility due to endometriosis [8], [30]. As noted above for marital status and testosterone (Figure 5A), *Comamonas testosteroni* TA441 is known to degrade testosterone [27], while an unnamed *Comamonas* species was associated with oral contraceptives (progesterone derivatives) here (Figures 5G and 6). Moreover, oral contraceptives exhibited a positive correlation with plasma homocitrulline (Figure 5G). These results would be worth investigating in large longitudinal cohorts.

Menstrual phases are known to influence the microbiota in the female reproductive tract [8], [22]. We confirmed in our cross-sectional cohort that *L. iners* was relatively more abundant in the proliferative phase (*i.e.*, after menses and before ovulation) than in the secretory phase (*i.e.*, after ovulation and before menses, waiting for implantion of embryos), while *L. crispatus* was relatively more abundant in the secretory phase than in the proliferative phase, coinciding with dynamics in progesterone as well as threonine and arginine (Figure 5H, Figure S7). White blood cell (WBC) counts also recovered after menses (Figure 5H). These results are consistent with the days after menses being more susceptible to BV relapse and a more stable vagino-cervical microbiota during or after ovulation [22]. Individuals with self-reported regular periods showed negative correlations with *Lactobacillus vaginalis*, *L. johnsonii*, and *Weissella* species, and lower levels of plasma androstenedione, testosterone, and serum LDL (Figure 6; Table S6). Women with a heavier flow showed relatively more abundant *Propionibacterium acnes* in the cervical sample, as well as higher plasma manganese (Mn) and cobalt (Co) levels, but reported less problems of constipation and alopecia (Figure 6; Table S6). For the gut microbiome, we have recently showed association between gut microbial functional potential for metabolism of secondary bile acids and frequency of defecation [31]. Consistent with the association with a heavier flow, *P. acnes* has been found to be higher in the endometrium in the secretory phase than the proliferative phase in our previous study on surgical samples [8], and has previously been identified in the placenta and cultured from the follicular fluid [32], [33], [34], [35]. Many women experience dysmenorrhea during menses (No: *n* = 84, Slight: *n* = 360, Serious: *n* = 68) (Table S1). Individuals with dysmenorrhea were enriched for Pseudomonadales, *Acinetobacter*, and Moraxellaceae, while lower in the plasma level of histidine (Figure 5I), consistent with these bacteria encoding histidine decarboxylases to convert histidine into histamine [36]. Individuals with dysmenorrhea preferred spicy food instead of plain food (Figure 5I; Table S6). Thus, every aspect of the menstrual cycle could be seen in the vagino-cervical microbiome, together with measurements of hormones, amino acids, and rare elements in circulation.

### Association between multi-omes

Integrated association network using a wisdom of crowds approach [37] also revealed interesting patterns (Spearman’s correlation, random forest, and linear regression, with arcsin square root-transform for the microbiome profiles) (Figure 6; Table S6). Psychological questionnaire data were available from the first cohort (Figure 1). The relative abundance of *G. vaginalis* was negatively associated with sleep and diet assessment, while positively associated with plasma levels of mercury (Hg) and vitamin E (Figure 6; Table S6). Note that the cerebrospinal fluid flows from brain arteries to veins during deep sleep [38]. TTV (*Anellovirus* genus), better known for its immune surveillance function in the serum [39], was found here in cervical samples to be positively associated with oral contraceptives, negative emotions, and pores on the forehead, and negatively associated with WBC counts, corticosterone, and 11-deoxycortisol (Figure 6; Table S6). *L. iners* was negatively correlated with spontaneous abortion, days since last menstrual bleeding, condom usage, and the plasma concentration of vitamin A, and positively correlated with the plasma concentrations of hemoglobin and alanine (Figure 6; Table S6).

While lacking significant associations with the vagino-cervical microbial composition, the immune repertoire data, especially TRBV7.3 and TRBV7.4, showed multiple associations with functional pathways in the vagino-cervical microbiome, such as purine and pyrimidine metabolism, and synthesis of branched-chain amino acids, histidine, and arginine (Figure S8). Red blood cell (RBC) counts, plasma vitamin A levels, and plasma hydroxyl vitamin D levels were negatively associated with CDP-diacylglycerol biosynthesis pathways, consistent with the presence of diacylglycerol kinase in lactobacilli with anti-inflammatory functions [40], [41]. Fecal *Coprococcus comes*, a bacterium previously reported to associate with cytokine response to *C. albicans* [42], was seen here to be associated with isoleucine pathways in the vagino-cervical microbiome (Figure S8). Plasma levels of rare elements including arsenic (As) and mercury (Hg) also showed associations with functional pathways in the vagino-cervical microbiome. Both As and Hg were negatively associated with *de novo* synthesis, salvage, and degradation of purine. As negatively associated with the degradation of pyrimidine deoxyribonucleosides, Hg was negatively associated with biosynthesis of methionine and S-adenosyl-L-methionine (Figure S8). These results underscore the metabolic potential of the vagino-cervical microbiome, which should be sampled in addition to the fecal microbiome [31].

The second cohort had 263 salivary metagenomic shotgun data (Figure 1), which allowed us to explore the potential contribution from the oral microbiome. Oral *Eikenella corrodens* and unnamed *Comamonas* species were positively correlated with *G. vaginalis* in vagina (Table S7). *Scardovia wiggsiae*, a bacterium previously reported to be associated with early childhood caries, showed a positive correlation with vaginal *Staphylococcus* species (Figure S9; Table S7). Oral *Treponema lecithinolyticum* showed a negative correlation with *Dialister micraerophilus* (Figure S9; Table S7). Oral *Prevotella baroniae* was negatively correlated with *L. crispatus* in vagina (Figure S9; Table S7). The *NimI* gene, which exhibits high-level resistance to metronidazole, was previously reported to be intrinsic to *P. baroniae* [43].

We next analyzed the integrated association network between multi-omes in the second cohort and confirmed associations that were observed in both cohorts (Figure S9; Table S8). Patterns consistent with the initial cohort could be identified, such as negative correlations between pregnancy history, current breastfeeding, and lactobacilli (*L. crispatus*, *L. jensenii*, *L. iners*, and *L. vaginalis*), higher dehydroepiandrosterone, androstenedione, and testosterone with *L. crispatus* (Figure S9; Table S8). *Prevotella buccalis* and *Prevotella timonensis* were negatively correlated with the number of vaginal deliveries and the physical fitness test score, but positively correlated with the number of cesarean sections. *Finegoldia magna* was negatively correlated with vitamin B1 and Hg; *U. parvum* was positively correlated with arginine, serine, and threonine, while negatively correlated with 25-hydroxy vitamin D, 17α-hydroxyprogesterone, and carnosine. *S. agalactiae* was negatively correlated with vitamin E, creatinine, and plasma concentration of hemoglobin. *P. bivia* was negatively correlated with 17α-hydroxyprogesterone, 11-deoxycorticosterone, progesterone, and creatinine*.* Individuals who enriched for *L. iners* ranked better in wrinkles and red area on the forehead*.* Both *A. vaginae* and *G. vaginalis* were positively correlated with the numbers of RBCs (Figure S9; Table S8).

A total of 779 volunteers (51.99% from the initial cohort, 48.01% from the second cohort) had corresponding fecal metagenomic shotgun data. Among vaginal-cervical microbes, *L. crispatus* showed the strongest positive correlation with fecal microbes (pair-wise associations for all), which was also *L. crispatus* (the initial cohort, rho = 0.386, *P* = 9.47E–15; the second cohort rho = 0.326, *P* = 2.24E–11) (Figure 6, Figure S9; Table S8). Fecal *L. crispatus* was also positively correlated with *L. vaginalis* in the cervical microbiome (Table S8). The BV species *P. bivia* was negatively associated with fecal *Butyricimonas* species and *Clostridia* species, while positively associated with fecal *P. bivia* (the initial cohort, rho = 0.115, *P* = 0.021; the second cohort, rho = 0.097, *P* = 0.059) (Figure S9; Table S8). We were able to assemble *P. bivia* from one fecal sample, and sequences from its corresponding vagino-cervical sample also supported the genome (Figure S10A). On the other hand, *L. crispatus* could be assembled from vagino-cervical data, while its coverage in fecal metagenome was typically low (Figure S10B). Fecal *Porphyromonas somerae* was positively correlated with *P. timonensis* in the vagino-cervical microbiome in both cohorts (Table S8). More cases would need to be followed to conclude whether cervical *L. crispatus* translocates to the gut, and whether fecal *P. bivia* translocates to the reproductive tract, or *vice versa*. Together, we summarized the associated features for the major species in the vagino-cervical microbiome (Table 1), which could be further investigated in future cohorts.

**Table 1:** **Summary the associated features for the major species in the vagino-cervical microbiome**

Bacterium	Associated feature	Associated feature type	Potential relevance
*Lactobacillus crispatus*	Higher in the secretory phase *vs.* the proliferative phase	Menstruation	Greater risk of BV resurgence after menses
	Single, or without previous pregnancy, or without previous delivery	Marriage/pregnancy/delivery	No such information available from iHMP when comparing ethnic groups [25]
	Not currently breastfeeding	Breastfeeding	Better recovery of the microbiome after delivery
	Vaginal sexual debut at younger ages	Sexual debut	Causal mechanism needed, *e.g.*, testosterone associated with *L. crispatus* has been associated with life-time number of sexual partners
	Less UV spots on forehead and cheeks	Facial skin imaging	
	Higher dehydroepiandrosterone, androstenedione, aldosterone, and testosterone	Hormones	
	Higher β-alanine	Amino acids	
	Higher fecal *Lactobacillus crispatus*	Fecal microbiome	
*Lactobacillus iners*	Without previous pregnancy, or lack of spontaneous abortion	Pregnancy	Detected in placenta, although controversial [10], [32], [71]
	Higher dehydroepiandrosterone and corticosterone	Hormones	
	Not currently breastfeeding	Breastfeeding	Better recovery of the microbiome after delivery
	Higher in the proliferative phase *vs.* the secretory phase	Menstruation	
	Oral contraceptive instead of condom	Contraception	HIV susceptibility in African countries [72]
	Less wrinkles on forehead and spots on cheeks	Facial skin imaging	Early, more effective treatment for a young look
	Higher alanine	Amino acids	
	Higher plasma concentrations of hemoglobin	Routine blood test	
	Lower 25-hydroxy vitamin D3	Vitamin	
*Lactobacillus gasseri*	Condom usage	Contraception	Contraception options
*Lactobacillus jensenii*	Without previous pregnancy	Pregnancy	
	Not currently breastfeeding	Breastfeeding	Better recovery of the microbiome after delivery
	Higher fecal *Haemophilus* species in individuals of the *Lactobacillus jensenii* type than in individuals of the *Lactobacillus crispatus* and *Atopobium vaginae* types	Fecal microbiome	
	Less spots on the cheek than women with *Lactobacillus crispatus* type	Facial skin imaging	
*Lactobacillus vaginalis*	Lower plasma androstenedione and testosterone	Hormones	
	Lower serum LDL	Routine blood test	
	Without previous pregnancy	Pregnancy	
	Not currently breastfeeding	Breastfeeding	Better recovery of the microbiome after delivery
	Young age	Age	
	Irregular menstruation	Menstruation	
*Gardnerella vaginalis*	RDW-SD	Routine blood test	
	Higher toughness assessment, psychological elasticity	Mental health conditions	
*Prevotella bivia*	Vaginal douching	Vaginal douching	BV or other infection
	Lower grip strength score	Fitness training	Enhance physical fitness to improve the microbiome
	Higher fecal *Prevotella bivia*	Fecal microbiome	
	Lower creatinine	Routine blood test	
	Lower 17α-hydroxyprogesterone, 11-deoxycorticosterone, and progesterone	Hormones	
*Prevotella timonensis*	Lower creatinine	Routine blood test	
	Number of caesarean sections instead of vaginal deliveries	Delivery	Benefits of vaginal deliveries
	Lower comprehensive score of physical fitness test, lower vertical jump score	Fitness training	Enhance physical fitness to improve the microbiome
*Prevotella buccalis*	Number of caesarean sections instead of vaginal deliveries	Delivery	Benefits of vaginal deliveries
	Lower comprehensive score of physical fitness test, lower vertical jump score	Fitness training	Enhance physical fitness to improve the microbiome
	Shorter menstrual period	Menstruation	
*Comamonas testosteroni*	Without marriage	Marriage	
	Dysmenorrhea	Menstruation	Effective treatment for dysmenorrhea
	Not oral probiotics within 1 month	Probiotics	Benefits of oral probiotics
*Streptococcus agalactiae*	Longer menstrual period	Menstruation	
	Lower vitamin E	Vitamin	
	Lower creatinine and plasma concentrations of hemoglobin	Routine blood test	
*Ureaplasma parvum*	More serious problem of porphyrins and texture on the forehead	Facial skin imaging	Early, more effective treatment for a young look
	Higher arginine, serine, and threonine; lower carnosine	Amino acids	
	Lower 25-hydroxy vitamin D	Vitamin	
	Lower 17α-hydroxyprogesterone	Hormones	
	Oral contraceptives	Contraception	Risk of oral contraceptives
*Propionibacterium acnes*	Heavier flow in menstrual period	Menstruation	Bacterium from endometrium and fallopian tube [8]
	Self-reported bacterial vaginosis	BV	BV risk
*Dialister micraerophilus*	Heavier flow in menstrual period	Menstruation	Phage or other treatment if really necessary
	Red area on the forehead	Facial skin imaging	Early, more effective treatment for a young look
	Lower creatinine	Routine blood test	
	Lower grip strength score	Fitness training	Muscle training

*Note*: BV, bacterial vaginosis; iHMP, integrative Human Microbiome Project; UV, ultraviolet; HIV, human immunodeficiency virus; LDL, low density lipoprotein; RDW-SD, red cell distribution width-standard deviation.

## Discussion

As the largest metagenomic shotgun study for the vagino-cervical microbiome, our data revealed less known subtypes for the vagino-cervical microbiota, as well as detecting fungal and viral sequences. Our multi-omic data could help target efforts aimed at promoting a healthy reproductive tract microbiota and offering better advices for mothers from pregnancy to recovery, as well as preventing infections from viruses such as HIV and HPV. For example, whether vitamin D supplementation should be considered in African countries to rise *L. crispatus*, as investigated recently in a cohort of pregnant women [44]. Gut probiotics such as *Lactobacillus casei* and *Bifidobacterium longum* have been reported to increase vitamin D in ovariectomy-induced mice model of osteoporosis [45], and our results imply that similar bacteria in the vagina might also be related to vitamin D metabolism.

During the course of the initial review process, publications from integrative Human Microbiome Project (iHMP) provided longitudinal data during pregnancy and showed relatively more *L. iners* compared to non-pregnant individuals even in women of African American history [16], [23], [25], [46]. In our data, the *L. iners vs. L. crispatus* shift was apparent in women with past pregnancy, and interestingly, *L. iners* showed a negative association with the number of spontaneous abortions (Figure 6; Table 1). *L. iners* and other lactobacilli have been detected in the placenta [10], [11], [32], [47], [48], amniotic fluid, nasal, and pharyngeal sites [49], [50]. Aldosterone, a major mineralocorticoid for which we observed an association with potentially beneficial bacteria in the gut microbiome in another study [31], was positively associated with *L. crispatus* in the vagino-cervical microbiome; on the other hand, a precursor for aldosterone, corticosterone, was positively associated with *L. iners* (Figure 6; Table 1). How vitamin D and hormone metabolism might impact the vagino-cervical microbiome would require further studies. How the uterus and the microbiome recover during breastfeeding is also of interest for both the mother and future children [16], [51]. *S. anginosus* has been shown to be more abundant in the gut microbiome of individuals with atherosclerotic cardiovascular diseases [52], and here we tentatively observed young women who harbored > 50% *S. anginosus* in cervical samples showing higher plasma creatinine and lower 17α-hydroxyprogesterone (Figure 2), which might relate to preterm birth [10]. The vagino-cervical microbiome of postmenopausal women revealed a myriad of viral and bacterial species (Figure S5), while fungal growth might be unfavorable both due to high pH and lack of glycans. While a focus in the field of the female vaginal microbiota has been infection and preterm birth, our data highlight major aspects of importance for female health that are worth further investigations for women in the modern world.

Samples from multiple body sites (peritoneal fluid from the pouch of Douglas, fallopian tubes, endometrium, cervical mucus, and two vaginal sites) have been analyzed from volunteers with benign conditions such as hysteromyoma, adenomyosis, and endometriosis [8], [9]. Here we were only able to sample the vagino-cervical microbiome in this healthy cohort. Interestingly, our cohort generally lacked bacteria known for BV (*e.g.*, studies from Serrano et al. [25] and Fettweis et al. [46]) and also contained less *Prevotella* compared to some of the surgically sampled individuals. In the postmenopausal samples, some of the bacteria previously reported in the upper reproductive tract [8], [9], [53] and involved in degradation of hormones [54], *e.g.*, *Pseudomonas* spp., could be seen in the vagino-cervical microbiome, while species of disease relevance such as *A. vaginae* and *Porphyromonas* in endometrial cancer would require more evidence [12].

Sampling of the cervix with a cytobrush by experienced doctors allowed the analyses of the microbiome that is generally lactobacilli-dominated like the vaginal sites of the lower reproductive tract [8], [9]. Cervix samples could also reflect the individual-specific continuum of the microbiome from the upper reproductive tract including the peritoneal cavity [8], [9]. We observed here that the association with the fecal microbiome was limited, and the notion of reservoirs in the intestine or other sites for vagino-cervical bacteria such as *L. crispatus* and *P. bivia* would need further investigation, especially in light of individual differences in the number of CD4^+^ T cells in mucosal sites [17], [55]. We observed other interesting associations between different species potentially involved in immune modulation (Figure 6). The plasma metabolites and T cell receptor types associated with the vagino-cervical microbiome were distinct from those associated with the fecal microbiome [31]. Vaginal *Prevotella* could induce more CD4^+^ T cells [17], but the *Prevotella copri* was not the dominant gut species of *Prevotella* species which may compete with *Bacteroides* spp. [52]. The vagino-cervical microbiome also better predicted facial skin features compared to the fecal microbiome [31], perhaps due to a clearer pattern of hormone and immune signatures. The associations with physical fitness tests and self-reported physical activities were, however, less prominent in the vagino-cervical microbiome compared to the fecal microbiome [31], as the changes due to pregnancy, delivery, and breastfeeding may not be easily modifiable with physical activity. Interesting associations were identified between the vagino-cervical microbiome and physical fitness test results, *e.g.*, *P. bivia* was negatively associated with hand grip strength, and *P. buccalis*, *Prevotella disiens*, and *Peptoniphilus harei* were negatively associated with vertical jump score. As a densely populated microbiota other than the distal gut, the vagino-cervical microbiota has the potential to reflect or even influence physiology elsewhere in the human body.

## Materials and methods

### Initial study cohort

As the first time point for the vagino-cervical microbiome of the 4D-SZ cohort, 516 Chinese volunteers joined from May to July during an annual physical examination in 2017. Baseline characteristics of the cohort are shown in Table S1.

### The second cohort

An independent cohort of 632 Chinese volunteers were recruited from May to July during an annual physical examination in 2018. 2018 data for 4D-SZ volunteers who were already included in the initial cohort were excluded and will be published in a future study. The collection procedures of samples and multi-omic data were similar to that in the initial cohort. In addition, salivary samples were collected only in this cohort. Baseline characteristics of the cohort are shown in Table S1.

### Demographic data collection

During physical examination, the volunteers received three kinds of online questionnaires. 1) The female life history questionnaire contained pregnancy and delivery histories, menstrual phases, sexual activity, and contraceptive methods. 2) The lifestyle questionnaire contained age, disease history, and eating and exercise habits. 3) The psychological questionnaire contained the evaluation of irritability, dizziness, frustration, fear, appetite, self-confidence, and resilience (Table S1).

### Sample collection

Cervical samples were collected and smeared in the Flinders Technology Associates (FTA) cards by doctor during gynecological examination. Fecal samples and salivary samples were self-collected by volunteers. Cervical samples, fecal samples, and salivary samples were stored at −80 °C for metagenomic shotgun sequencing. The overnight fasting blood samples were drawn from a cubital vein of volunteers by doctor.

### DNA extraction and metagenomic shotgun sequencing

DNA extraction of cervical samples and fecal samples was performed as described [8], [56]. Metagenomic shotgun sequencing was performed on the BGISEQ-500 platform, which is highly comparable to Illumina HiSeq platforms in metagenomic and other sequencing applications [9], [57], [58], [59]. The 50 bp of single-end reads for cervical samples collected in the initial study cohort, and on an average of 208.76 million raw reads were sequenced for each sample (Table S2); the 100 bp of single-end reads for fecal samples collected in the initial study cohort, and on an average of 85.63 million raw reads were sequenced for each sample (Table S2); the 100 bp of paired-end reads for cervical samples, fecal samples, and salivary samples collected in the second cohort, and on an average of 158.91 million raw reads for cervical samples, 151.69 million raw reads for salivary samples, and 148.26 million raw reads for fecal samples were sequenced for each sample, respectively (Table S2). Quality control and alignment to GRCh38 were performed as previously described [9], [57].

### Ultra high pressure liquid chromatography-mass spectrometry quantification of amino acids

40 µl of plasma was deproteinized with 20 µl of 10% (w/v) sulfosalicylic acid (Sigma) containing internal standards, and then 120 µl of aqueous solution was added. After centrifuged, the supernatant was used for analysis. The analysis was performed by ultra high pressure liquid chromatography (UHPLC) coupled to an AB Sciex Qtrap 5500 MS (AB Sciex, Los Angeles, CA) with the electrospray ionization (ESI) source in positive ion mode. A Waters ACQUITY UPLC HSS T3 column (1.8 µm, 2.1 mm × 100 mm) was used for amino compound separation with a flow rate at 0.5 ml/min and column temperature of 55 °C. The mobile phases were pahse A [water containing 0.05% heptafluorobutyric acid and 0.1% formic acid (v/v)] and phase B [acetonitrile containing 0.05% heptafluorobutyric acid and 0.1% formic acid (v/v)]. The gradient elution was 2% B kept for 0.5 min, then changed linearly to 10% B during 1 min, continued up to 35% B in 2 min, increased to 95% B in 0.1 min, and maintained for 1.4 min. Multiple Reaction Monitoring (MRM) was used to monitor all amino compounds. The mass parameters were as follows: curtain gas flow rate was 35 l/min; Collision Gas (CAD) was medium; Ion Source Gas 1 (GS 1) flow rate was 60 l/min; Ion Source Gas 2 (GS 2) flow rate was 60 l/min; IonSpray Voltage (IS) was 5500 V and temperature was 600 °C. All amino standard reagents were purchased from Sigma-Aldrich (St. Louis, MO) and Toronto research chemical (TRC).

### UHPLC-MS quantification of hormones

250 µl of plasma was diluted with 205 µl of sterile water. For solid-phase extraction (SPE) experiments, hydrophilic lipophilic balance (HLB, Waters) was supplemented with 1.0 ml for each of dichloromethane, acetonitrile, and methanol, and was equilibrated with 1.0 ml of water. The pretreated plasma sample was extracted using gravity. Clean up was accomplished by washing the cartridges with 1.0 ml of 25% methanol in water. After drying under vacuum, samples on the cartridges were eluted with 1.0 ml of dichloromethane. The eluted extract was dried under nitrogen, and the residual was dissolved with 25% methanol in water and transferred to an autosampler vial prior to UHPLC-MS analysis. The analysis was performed by UHPLC coupled to an AB Sciex Qtrap 5500 MS (AB Sciex) with the atmospheric pressure chemical ionization (APCI) source in positive ion mode. A Phenomone Kinetex C18 column (2.6 µm, 2.1 mm × 50 mm) was used for steroid hormone separation with a flow rate at 0.8 ml/min and column temperature of 55 °C. The mobile phases were phase A (water containing 1 mM ammonium acetate) and phase B (methanol containing 1 mM ammonium acetate). The gradient elution was 25% B kept for 0.9 min, then changed linearly to 40% B during 0.9 min, continued up to 70% B in 2 min, increased to 95% B in 0.1 min, and maintained for 1.6 min. MRM was used to monitor all steroid hormone compounds. The mass parameters were as follows: curtain gas flow rate was 35 l/min; CAD was medium; GS 1 flow rate was 60 l/min; GS 2 flow rate was 60 l/min; Nebulizer Current (NC) was 5 and temperature was 500 °C. All steroid hormone profiling compound standards were purchased from Sigma-Aldrich, TRC, Cerilliant, and Dr. Ehrenstorfer.

### Inductively coupled plasma-mass spectrometry quantification of trace elements

200 µl of whole blood was transferred into a 15-ml polyethylene tube and diluted 1:25 with a diluent solution consisting of 0.1% (v/v) Triton X-100, 0.1% (v/v) HNO_3_, 2 mg/l AU, and internal standards (20 µg/l). The mixture was sonicated for 10 min before inductively coupled plasma-mass spectrometry (ICP-MS) analysis. Multi-element determination was performed on an Agilent 7700x ICP-MS (Agilent Technologies, Tokyo, Japan) equipped with an octupole reaction system (ORS) collision/reaction cell technology to minimize spectral interferences. The continuous sample introduction system consisted of an autosampler, a quartz torch with a 2.5-mm diameter injector with a Shield Torch system, and a Scott double-pass spray chamber and nickel cones (Agilent Technologies). A glass concentric MicroMist nebuliser (Agilent Technologies) was used for the analysis of diluted samples.

### Ultra pressure liquid chromatography-mass spectrometry quantification of water-soluble vitamins

200 µl of plasma was deproteinized with 600 µl of methanol (Merck), water, and acetic acid (9:1:0.1) containing internal standards, thiamine-(4-methyl-13C-thiazol-5-yl-13C3) hydrochloride (Sigma-Aldrich), levomefolic acid-13C, d3, riboflavin-13C,15N2, 4-pyridoxic acid-d3, and pantothenic acid-13C3,15N hemi calcium salt (Toronto Research Chemicals). 500 µl of supernatant was dried under a nitrogen flow. 60 µl of water was added to the residues, and then vortexed. The mixture was centrifuged and the supernatant was used for analysis. The analysis was performed by ultra pressure liquid chromatography (UPLC) coupled to a Waters Xevo TQ-S Triple Quad MS (Waters) with the ESI source in positive ion mode. A Waters ACQUITY UPLC HSS T3 column (1.7 µm, 2.1 mm × 50 mm) was used for water-soluble vitamin separation with a flow rate at 0.45 ml/min and column temperature of 45 °C. The mobile phases were phase A (0.1 % formic acid in water) and phase B (0.1% formic acid in methanol). The following elution gradient was used: 0–1 min, 99% A; 1–1.5 min, 99%–97% A; 1.5–2 min, 97%–70% A; 2–3.5 min, 70%–30% A; 3.5–4 min, 30%–10% A; 4–4.8 min, 10% A; 4.9–6 min, 99% A. MRM was used to monitor all water-soluble vitamins. The mass parameters were as follows: the capillary voltages of 3000 V and source temperature of 150 °C were adopted. The desolvation temperature was 500 °C. The CAD flow rate was set at 0.10 ml/min. The cone gas and desolvation gas flow rates were 150 l/h and 1000 l/h, respectively. All water-soluble vitamin standards were purchased from Sigma-Aldrich.

### UPLC-MS quantification of fat-soluble vitamins

250 µl of plasma was deproteinized with 1000 µl of methanol and acetonitrile (v/v, 1:1) (Fisher Chemical) containing internal standards, all-trans-Retinol-d5, 25-HydroxyVitamin-D2-d6, 25-HydroxyVitamin-D3-d6, vitamin K1-d7, and α-Tocopherol-d6 (Toronto Research Chemicals). 900 µl of supernatant was dried under a nitrogen flow. 80 µl of 80% acetonitrile was added to the residues, and then vortexed. The mixture was centrifuged, and the supernatant was used for analysis. The analysis was performed by UPLC coupled to an AB Sciex Qtrap 4500 MS (AB Sciex) with the APCI source in positive ion mode. A Waters ACQUITY UPLC BEH C18 column (1.7 µm, 2.1 mm × 50 mm) was used for fat-soluble vitamin separation with a flow rate at 0.50 ml/min and column temperature of 45 °C. The mobile phases were phase A (0.1 % formic acid in water) and phase B (0.1% formic acid in acetonitrile). The following elution gradient was used: 0–0.5 min, 60% A; 0.5–1.5 min, 60%–20% A; 1.5–2.5 min, 20%–0% A; 2.5–4.5 min, 0% A; 4.5–4.6 min, 0%–60% A; 4.6–5.0 min, 60% A. MRM was used to monitor all fat-soluble vitamins. The mass parameters were as follows: curtain gas flow rate was 30 l/min; CAD was medium; GS 1 flow rate was 40 l/min; GS 2 flow rate was 50 l/min; NC was 5 and the temperature was 400 °C. All fat-soluble vitamin standards were purchased from Sigma-Aldrich and TRC.

### Sequencing of the T-cell receptor β complementarity-determining region 3 immune repertoire

10 ml of whole blood was centrifuged at 3000 r/min for 10 min, and then 165 µl of buffy coat was obtained to extract DNA using MagPure Buffy Coat DNA Midi KF Kit (Magen, China). The DNA was sequenced on the BGISEQ-500 platform using 200 bp single-end reads. Data processing was performed using Immune IMonitor [60].

### Medical parameters

All the volunteers were recruited during the physical examination. The medical test included blood tests, urinalysis, and routine examination of cervical secretion. InBody was measured by InBody Analyzer (InBody Co., Ltd). All the medical parameters were measured by the physical examination center and shown in Table S1.

### Facial skin features

The volunteers were required to clean their face and use no makeup after they got up in the morning. The volunteer’s frontal face was photographed by VISIA-CRTM imaging system (Canfield Scientific, Fairfield, NJ) equipped with chin supports and forehead clamps that fix the face during the photographing process and maintain a fixed distance between the volunteers and the camera at all times. Eight indices were obtained including spots, pores, wrinkles, texture, UV spots, porphyrins, brown spots, and red area from the cheek and forehead, respectively (Table S1). The percentile for each index was calculated based on the index value ranked in the age-matched database (Table S1). The higher the percentile of an index, the better the facial skin appears.

### Physical fitness test

Eight kinds of tests were performed to evaluate the volunteers’ physical fitness condition (Table S1). Vital capacity was measured by HK6800-FH (Hengkangjiaye, China). Single-legged stance with eyes closed was measured by HK6800-ZL. Choice reaction time was measured by HK6800-FY. Grip strength was measured by HK6800-WL. Sit-and-reach score was measured by HK6800-TQ. Sit-ups were measured by HK6800-YW. Step index was measured by HK6800-TJ. Vertical jump was measured by HK6800-ZT. We got a measure value from each test. Then each measure value score was assigned grades A through E based on its corresponding age-matched database.

### Quality control, taxonomic annotation, and abundance calculation

The sequencing reads were quality-controlled by the overall accuracy (OA) control strategy as described previously [57]. Reads with length no short than 30 bp, seed OA higher than 0.9, and fragment OA higher than 0.8 were retained.

Host sequence contamination reads in fecal and salivary samples were removed using soap2.22 and hg19 index with parameters “-M 4 -l 30 -r 1 -m 200 -x 600 -v 8 -c 0.9”. As there were over 90% of human sequences in cervical samples, the stringent condition for removal of host sequences was used [9]. In short, after using the same SOAP2 process, the retained reads were mapped to hg19, hg38, and YH indexes by DeconSeq [61] and SNAP1.0 [62], respectively. Unaligned reads were retained for analysis.

Taxonomic assignment of the high-quality cervical metagenomic shotgun data and salivary metagenomic shotgun data was performed using MetaPhlAn2 [63].

Taxonomic assignment of the high-quality fecal metagenomic shotgun data was performed using the reference gene catalog comprising 9,879,896 genes [56]. Taxonomies of the fecal metagenomic linkage groups / metagenomic species were then determined from their constituent genes, as previously described [4], [64], [65].

The vaginal samples were hierarchically clustered using R base hcluster function with centroid linkage based on Euclidean distance in Figure 2A.

### Random forest on the influence of female life history factors

The factors in the female life history questionnaire were fitted against the relative abundances of taxonomic profiles (found in at least 10% of the samples) of the cervical samples using default parameters in the RFCV regression function from randomForest package in R. Female life history factors, except age (a continuous variable), are either dummy variables such as pregnancy history (yes, no) or frequency variables such as number of caesarean sections (0, 1, 2) (Table S1). In addition to comparing the predictive power across factors, we used regression model instead of classification model here. Spearman’s correlation between the measured value and the 5-fold cross-validation predicted value was calculated as a model performance metric. Then the key predictable factors were ranked. *P* value was obtained using permutation test (999 times).

### The global effect size between vagino-cervical microbiome and omic data

To evaluate the combined effect size of vagino-cervical microbiome on omic data, we used forward stepwise redundancy analysis of omic data lists on the relative abundances of taxonomic profiles in forward.sel function in the packfor package in R. This analysis provided a global *vs.* global association between any two omic datasets that maximizes the associations using the strongest predictive power of non-redundant predictors.

### The factors in each type of omes predicted by vagino-cervical microbiome

The factors in each type of omes were fitted against the relative abundances of taxonomic profiles (found in at least 10% of the samples) of the cervical samples using default parameters in the RFCV regression function from randomForest package in R. Omic data are a mix of dummy variables and continuous variables (Table S1). In addition to comparing the predictive power across factors, we used regression model instead of classification model here. Spearman’s correlation between the measured value and the 5-fold cross-validation predicted value was calculated as a model performance metric. Then the top 8 predictable factors in each type were ranked. *P* value was obtained using permutation test (999 times).

### Transformation of metagenomic shotgun profiles for composition data analysis

We normalized the microbiome data with arcsine square root transformation to make it less skewed for the downstream analysis (implemented in MaAsLin software [66]).

### Wisdom of crowds for robust network construction between vagino-cervical microbial species and multi-omic data

A new method for multi-omics analyses [37] was used to integrate the coefficient of linear regression, variance importance from randomForest, and Spearman’s correlation to construct omic flux networks, and then visualized the networks in Cytoscape. The details are as indicated below.

Step 1: Data processing. All categorical variables in multi-omic data were converted into continuous variables, and nominal variables were converted into dummy variables. Missing values were filled with median, and samples containing more than 70% missing variables were removed. The microbial species less than 10% in all the samples were also removed, as well as the near zero variable variables. For linear models, variables were normalized. Outliers were defined as outside of the 95% quartiles and removed.

Step 2: Method implementation. Random forest variable importance was used to identify the most important predictor variables [67]. RFCV regression function from randomForest package in R with default parameters was used to get the 5-fold average variable importance. We calculated the Spearman’s correlation with the cor.test function in base R software. For linear regression, we considered penalty regression to overcome the sparse and co-linear problem, cv.glmnet function from glmnet package in R was first used to figure out the best lambda parameter, and then bootstrapping glmnet with 0.632 re-sampling was performed 100 times to get the best lambda.

Step 3: Construction of robust networks. We kept first 5 average ranks for each target variable and retained edges with Spearman’s correlation *Q* value < 0.1. Then ggplot package in R was used to make barplot for some representative female life history factors (Figure 5). Cytoscape was also used to visualize the omic network (Figure 6).

The second cohort was analyzed using the same statistical method. Combining *P* value is computed using Edgington method from metap package in R. BH method was used to adjust the multiple test *P* value (*Q* value). The correlation is identified based on *Q* < 0.1 when a similar microbial distribution pattern shown in the initial study cohort and the second cohort.

### Association between microbiome pathways and multi-omes

Pathway profiles were calculated from the vagino-cervical metagenomic shotgun data using humann2. Spearman’s correlation was calculated between the relative abundance of each pathway and other numerical data collected. The R package heatmap was used for visualization. *Q* < 0.1 was considered as significant.

### Circular genome map of ***P. bivia*** and ***L. crispatus***

The high-quality reads of fecal and vaginal samples from two separate individuals with high amounts of *L. crispatus* or *P. bivia* were singled out to assemble using metaSPAdes (with the parameter “spades.py --meta -t 8 -m 50”). Then Blastn (with the parameter “blastn -word_size 16 -outfmt 6 -evalue 1e-10 -max_target_seqs 5000 -num_threads 8”) was used to extract the contigs of corresponding species. The read depth of the contigs was aligned to the RefSeq Database (2019-06-06) with the parameter “bwa mem -t 8”. The comparison of the vaginal and fecal assembled genomes from the same individual was visualized by BRIG (https://bmcgenomics.biomedcentral.com/articles/10.1186/1471–2164-12–402).

## Ethical statement

The study was approved by the Institutional Review Boards (IRB) at BGI-Shenzhen, China, and all participants provided signed informed consent at enrollment.

## Data availability

Metagenomic shotgun data for all samples and other relevant data reported in this study have been deposited in both CNGB Sequence Archive [68] of China National GeneBank DataBase (CNGBdb) [69] (CNSA: CNP0000287), and the Genome Warehouse [70] at the National Genomics Data Center, Beijing Institute of Genomics, Chinese Academy of Sciences / China National Center for Bioinformation (BioProject: PRJCA003712) which are publicly accessible at https://ngdc.cncb.ac.cn/gsa/.

## CRediT author statement

**Zhuye Jie:** Conceptualization, Methodology, Software, Visualization, Writing - original draft. **Chen Chen:** Conceptualization, Investigation, Visualization, Writing - original draft, Project administration. **Lilan Hao:** Investigation, Methodology, Formal analysis, Visualization, Writing - original draft. **Fei Li:** Formal analysis, Visualization. **Liju Song:** Investigation. **Xiaowei Zhang:** Writing - original draft. **Jie Zhu:** Formal analysis. **Liu Tian:** Formal analysis. **Xin Tong:** Investigation. **Kaiye Cai:** Investigation. **Zhe Zhang:** Writing - original draft. **Yanmei Ju:** Investigation. **Xinlei Yu:** Investigation. **Ying Li:** Investigation. **Hongcheng Zhou:** Investigation. **Haorong Lu:** Investigation. **Xuemei Qiu:** Investigation. **Qiang Li:** Investigation. **Yunli Liao:** Investigation. **Dongsheng Zhou:** Investigation. **Heng Lian:** Investigation. **Yong Zuo:** Investigation. **Xiaomin Chen:** Investigation. **Weiqiao Rao:** Investigation. **Yan Ren:** Investigation. **Yuan Wang:** Investigation. **Jin Zi:** Investigation. **Rong Wang:** Investigation. **Na Liu:** Investigation. **Jinghua Wu:** Investigation. **Wei Zhang:** Investigation. **Xiao Liu:** Investigation. **Yang Zong:** Investigation. **Weibin Liu:** Investigation. **Liang Xiao:** Supervision. **Yong Hou:** Supervision. **Xun Xu:** Supervision. **Huanming Yang:** Supervision. **Jian Wang:** Supervision. **Karsten Kristiansen:** Writing - review & editing. **Huijue Jia:** Conceptualization, Writing - review & editing, Supervision, Project administration. All authors have read and approved the final manuscript.

## Competing interests

The authors have declared no competing interests.

## Supplementary material

Supplementary Figure S1 **Representative vaginal microbiota types identified in 516 women**. **A**. The ratio of different vaginal-cervical microbiota types. The species whose relative abundance account for 50% or higher in an individual is defined as a community type, while all species accounting for less than 50% of the microbiota in an individual are collectively identified as a diverse community type. The microbiota types that represent less than 1% of the 516 individuals are collectively labeled as ‘Multi-subtypes’. The ratio and number of samples per microbiota type are labeled. **B**. Percentage of non-human sequences in the dominant vaginal-cervical types (except Multi-types). Wilcoxon ranked sum test is used to calculated the difference among the vaginal-cervical types. An asterisk denotes *P* value < 0.05, two asterisks denote *P* value < 0.01, and three asterisks denote *P* value < 0.001. The boxes denote the IQR between the first and third quartiles (25th and 75th percentiles, respectively), and the line inside the boxes denotes the median. The whiskers denote the lowest and highest values within 1.5 times of the IQR from the first and third quartiles, respectively. IQR, interquartile range.

Supplementary Figure S2 **The ratio of volunteers with breakdown experience in pregnancy history in the initial cohort**. The different colors represent for the volunteers who experienced 1–6 times of having the experience of pregnancy, delivery, caesarean section, and abortion. N/A means lack of this data.

Supplementary Figure S3 **Factors of female life history influencing the vagino-cervical microbiome in the second cohort**. Influence of female life history questionnaire entries on the vagino-cervical microbiome, ordered according to their 5-fold cross-validated random forest importance on the microbiome composition. X-axis (length of the bar) is the model performance measured as the Spearman’s correlation between the prediction and measurement. First column symbols after Y-axis label are 999 times permutation P value, second column symbols are adjusted P values for BH method (26 comparisons), “-” denotes value≥ 0.1, “+” denotes value < 0.1, “*” denotes value < 0.05, “**” denotes value < 0.01, “***” denotes value < 0.001. The red labels denote the phenotypes in the both cohorts.

Supplementary Figure S4 **Vagino-cervical microbiome of 137 post-partum women from two cohorts**. The microbial composition in each sample at the species level (**A**) and kingdom level (**B**) according to MetaPhlAn3 is shown. The dendrogram in (A) is a result of a centroid linage hierarchical clustering based on Euclidean distances between the microbial composition proportion. The bottom portion of the (A) illustrates months of breast-feeding by the time of sampling for each subject and the source of each sample, and the max marked by red which is 40 months in the bar. Due to months of breastfeeding is only available in the second cohort, the grey bars mean lack of this data. Purple, black, and red labels used in legend denote kingdom for bacteria, viruses, and eukaryote, respectively. **C**. The ratio of different vaginal-cervical microbiota types. The species whose relative abundance account for more than 50% in an individual are selected as an identified type. Species all account less than 50% of the microbiota in an individual is identified as diverse type. The ratio and number of samples per microbiota type are labeled.

Supplementary Figure S5 **Vagino-cervical microbiome of the postmenopausal women in the second cohort**. The microbial composition in each sample at the species level (**A**) and kingdom level (**B**) according to MetaPhlAn3 is shown. The dendrogram in (A) is a result of a centroid linage hierarchical clustering based on Euclidean distances between the microbial composition proportion. Purple, black, and red taxa labels used in legend denote kingdom for bacteria, viruses, and unclassified, respectively. **C**. The ratio of different vaginal-cervical microbiota types. The species whose relative abundance account for more than 50% in an individual are selected as an identified type. Species all account less than 50% of the microbiota in an individual is identified as diverse type. The ratio and number of samples per microbiota type are labeled.

Supplementary Figure S6 **Comparisons of species relative abundance between the longer and truncated reads**. The taxonomic profile from PE100, pseudo-SE100, and pseudo-SE50 are compared by truncating the paired reads of 45 postmenopausal samples in the second cohort. The relative abundance of the top 7 species in the samples are shown. X-axis in the three panels denote using the pseudo-SE50 (**A**), pseudo-SE50 (**B**), and pseudo-SE100 (**C**) mode, respectively. Y-axis in the three panels denote using the pseudo-SE100 (**A**), PE100 (**B**), and PE100 (**C**) mode, respectively. A blank spot denotes a sample. The fitting blue line is robust linear model regress one mode against another mode.

Supplementary Figure S7 **Shift of *L. crispatus* and *L. iners* showing significant difference during menstrual cycle in the initial study cohort**. The length of menstrual cycle of each woman is normalized to 28 days. The proliferative phase is from the end of the period to day 14, and the secretory phase is from the 15th to 28th day of a menstrual cycle. P value is calculated using Wilcoxon ranked sum test. The boxes denote the IQR between the first and third quartiles (25th and 75th percentiles, respectively), and the line inside the boxes denotes the median. The whiskers denote the lowest and highest values within 1.5 times the IQR from the first and third quartiles, respectively. IQR, interquartile range.

Supplementary Figure S8 **Association between functional pathways of the vagino-cervical microbiome and multi-omics in the initial study cohort**. Spearman’s correlation between pathway profiles according to HUMAnN2 and the numerical data from multi-omics. Only those associated significantly with at least one pathway are shown (“+” denotes *Q* value < 0.1, “*” denotes *Q* value < 0.05, “**” denotes *Q* value < 0.01).

Supplementary Figure S9 **Wisdom of the crowds for the association network between vagino-cervical microbial species and other multi-omic data in the second cohort**. Results from generalized linear model with penalty (cv.glmnet), random forest (RFCV), and Spearman’s correlation are integrated and then visualized in Cytoscape. Red lines for negative associations; cyan lines for positive associations.

Supplementary Figure S10 **Circular genome map of *P. bivia* (A) and *L. crispatus* (B) assembled from two separate individuals, including their fecal and vaginal samples**. The inner to outer circles show (i) the GC content (black); (ii) the GC skew (+/−): values > 0 (green) and values < 0 (purple); (iii) the sequencing depth with log10-transformed of vaginal sample: values > 0 (pink) and values < 0 (red); (v) the sequencing depth with log10-transformed of fecal sample: values > 0 (light blue) and values < 0 (blue); (vi) assembled contigs from vaginal metagenomic shotgun data; (vii) assembled contigs from fecal metagenomic shotgun data.
