# Supplementary figures and images for: Life History Recorded in the Vagino-cervical Microbiome Along with Multi-omes
Source: Genomics Proteomics Bioinformatics. 2021 Jun 9;20(2):304–21. doi: 10.1016/j.gpb.2021.01.005 (PMC9684086; doi:10.1016/j.gpb.2021.01.005)

**A**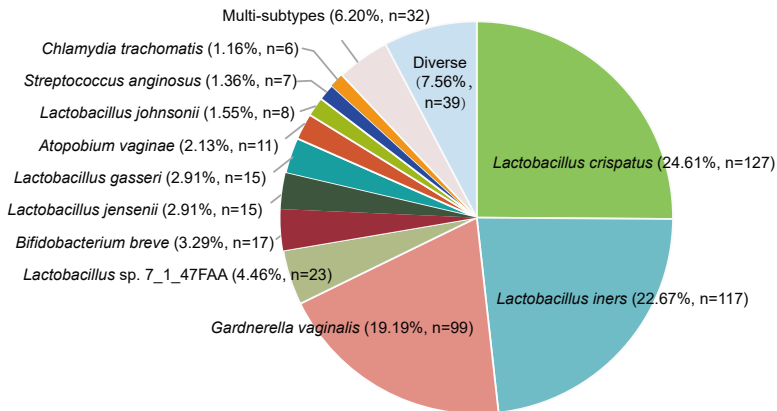**B**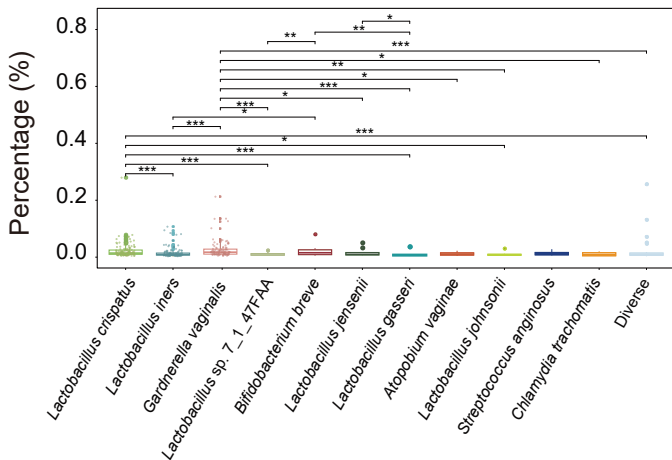

Supplement: Supplementary Figure S1 — Representative vaginal microbiota types identified in 516 women. A. The ratio of different vaginal-cervical microbiota types. The species whose relative abundance account for 50% or higher in an individual is defined as a community type, while all species accounting for less than 50% of the microbiota in an individual are collectively identified as a diverse community type. The microbiota types that represent less than 1% of the 516 individuals are collectively labeled as ‘Multi-subtypes’. The ratio and number of samples per microbiota type are labeled. B. Percentage of non-human sequences in the dominant vaginal-cervical types (except Multi-types). Wilcoxon ranked sum test is used to calculated the difference among the vaginal-cervical types. An asterisk denotes P value < 0.05, two asterisks denote P value < 0.01, and three asterisks denote P value < 0.001. The boxes denote the IQR between the first and third quartiles (25th and 75th percentiles, respectively), and the line inside the boxes denotes the median. The whiskers denote the lowest and highest values within 1.5 times of the IQR from the first and third quartiles, respectively. IQR, interquartile range. [file mmc1.pdf]

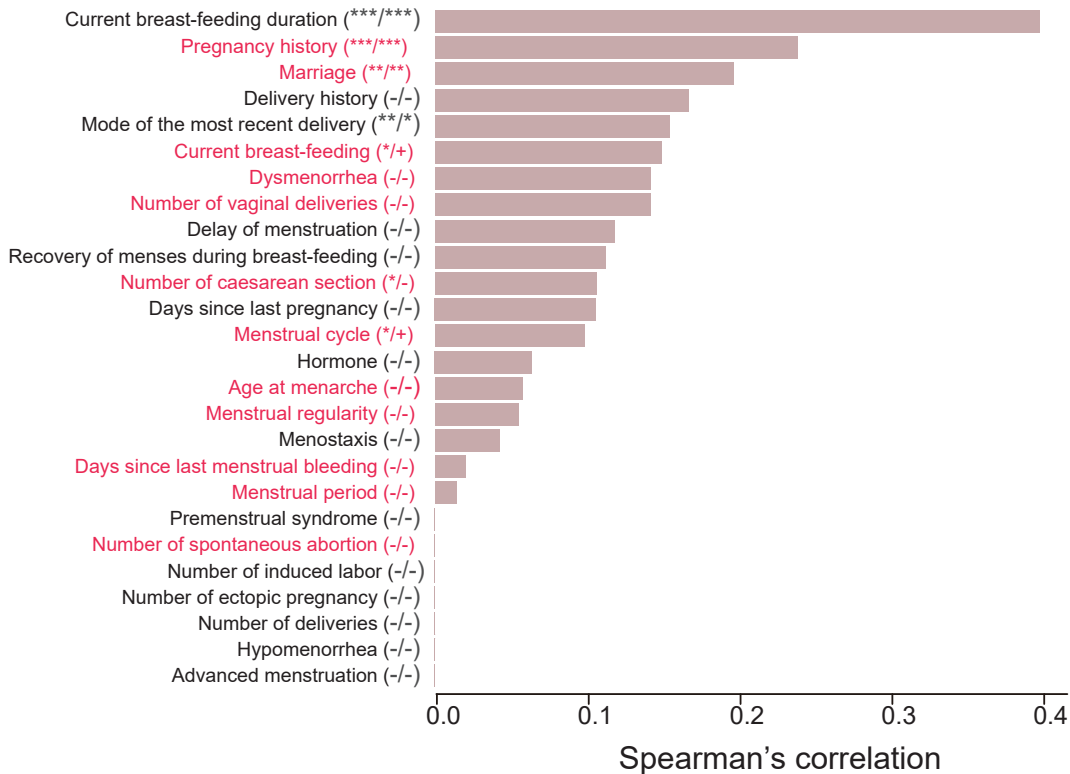

Supplement: Supplementary Figure S3 — Factors of female life history influencing the vagino-cervical microbiome in the second cohort. Influence of female life history questionnaire entries on the vagino-cervical microbiome, ordered according to their 5-fold cross-validated random forest importance on the microbiome composition. X-axis (length of the bar) is the model performance measured as the Spearman’s correlation between the prediction and measurement. First column symbols after Y-axis label are 999 times permutation P value, second column symbols are adjusted P values for BH method (26 comparisons), “-” denotes value≥ 0.1, “+” denotes value < 0.1, “*” denotes value < 0.05, “**” denotes value < 0.01, “***” denotes value < 0.001. The red labels denote the phenotypes in the both cohorts. [file mmc3.pdf]

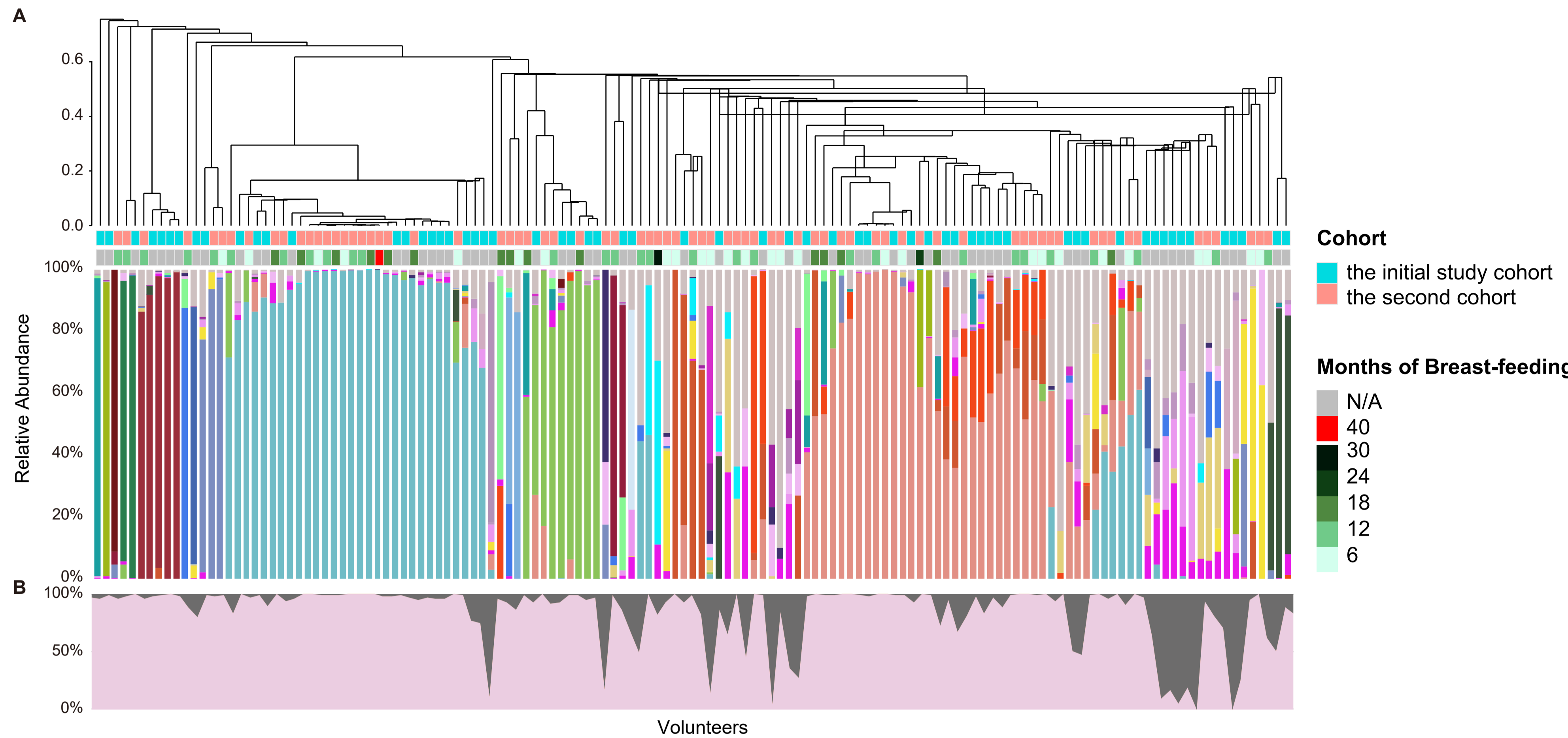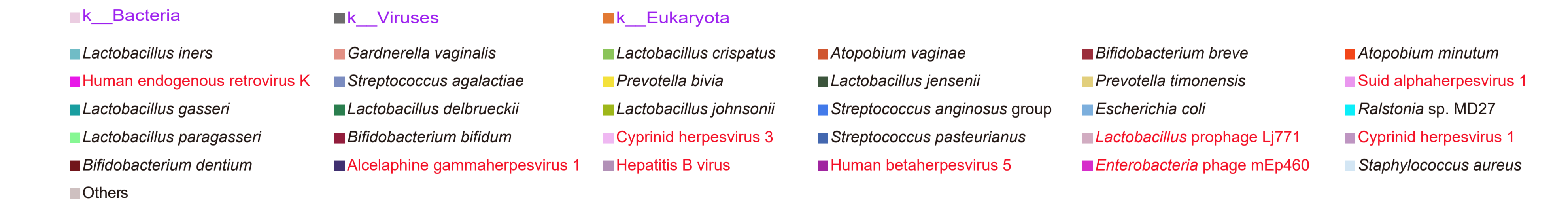

Supplement: Supplementary Figure S4 — Vagino-cervical microbiome of 137 post-partum women from two cohorts. The microbial composition in each sample at the species level (A) and kingdom level (B) according to MetaPhlAn3 is shown. The dendrogram in (A) is a result of a centroid linage hierarchical clustering based on Euclidean distances between the microbial composition proportion. The bottom portion of the (A) illustrates months of breast-feeding by the time of sampling for each subject and the source of each sample, and the max marked by red which is 40 months in the bar. Due to months of breastfeeding is only available in the second cohort, the grey bars mean lack of this data. Purple, black, and red labels used in legend denote kingdom for bacteria, viruses, and eukaryote, respectively. C. The ratio of different vaginal-cervical microbiota types. The species whose relative abundance account for more than 50% in an individual are selected as an identified type. Species all account less than 50% of the microbiota in an individual is identified as diverse type. The ratio and number of samples per microbiota type are labeled. [file mmc4.pdf]

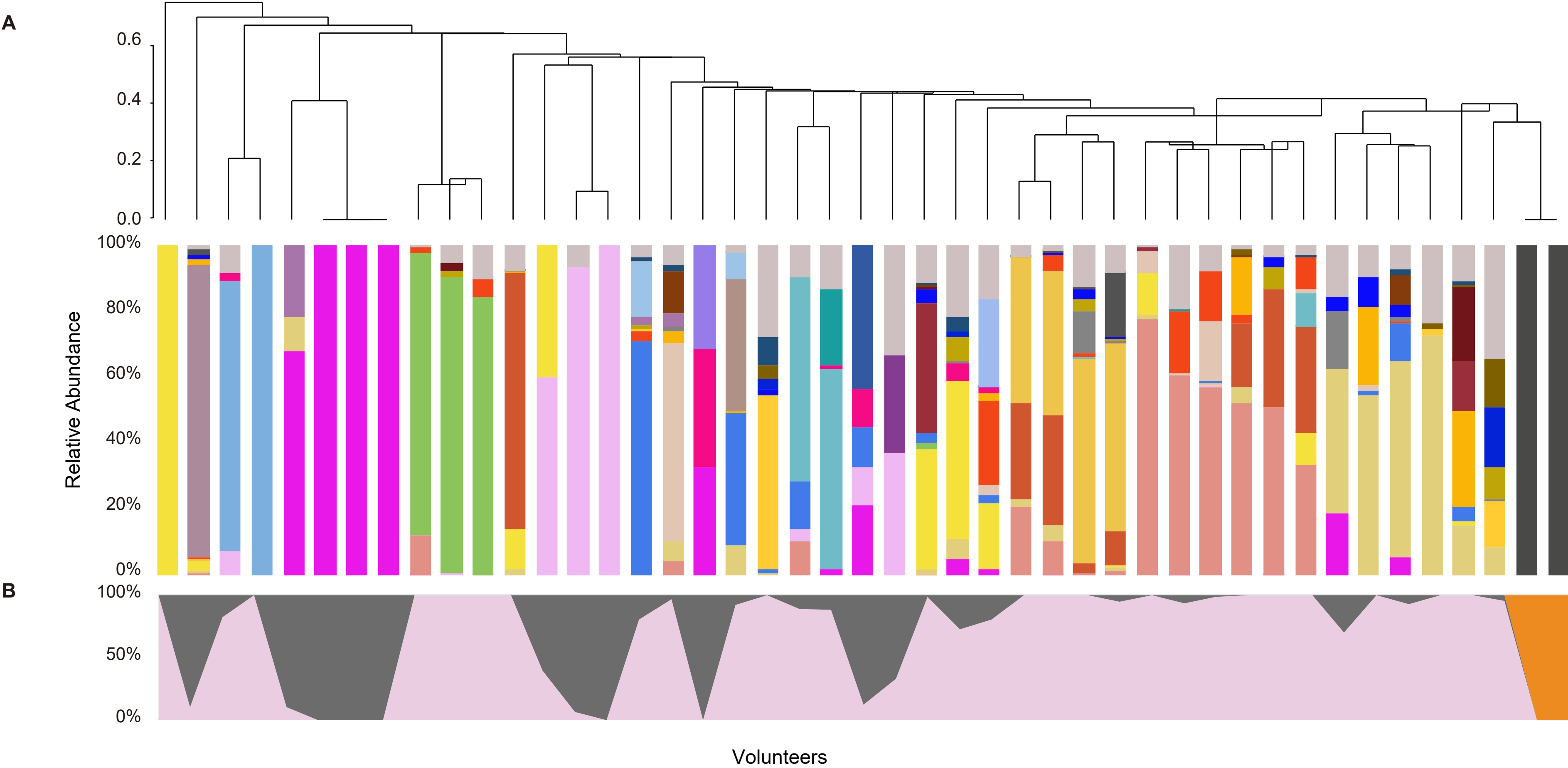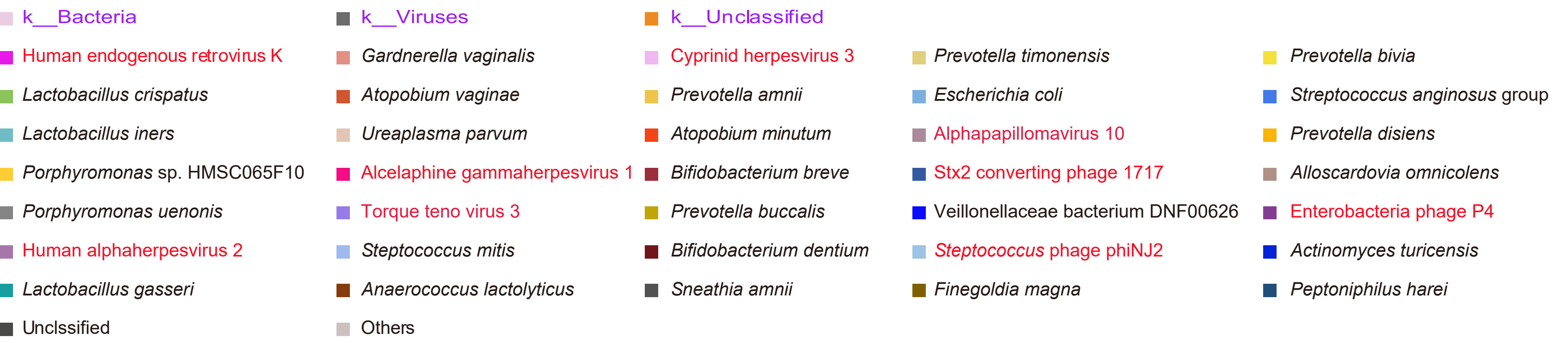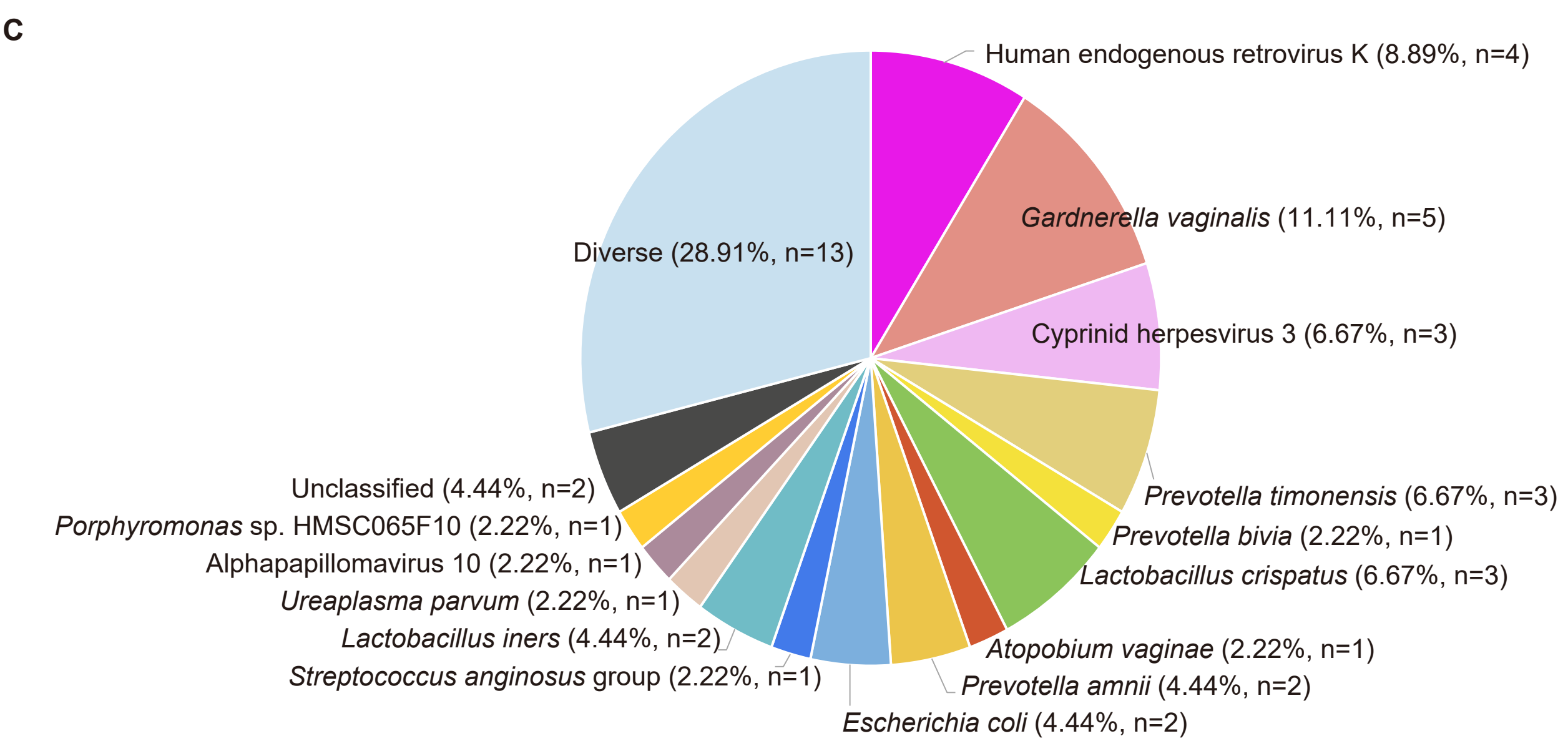

Supplement: Supplementary Figure S5 — Vagino-cervical microbiome of the postmenopausal women in the second cohort. The microbial composition in each sample at the species level (A) and kingdom level (B) according to MetaPhlAn3 is shown. The dendrogram in (A) is a result of a centroid linage hierarchical clustering based on Euclidean distances between the microbial composition proportion. Purple, black, and red taxa labels used in legend denote kingdom for bacteria, viruses, and unclassified, respectively. C. The ratio of different vaginal-cervical microbiota types. The species whose relative abundance account for more than 50% in an individual are selected as an identified type. Species all account less than 50% of the microbiota in an individual is identified as diverse type. The ratio and number of samples per microbiota type are labeled. [file mmc5.pdf]

**A**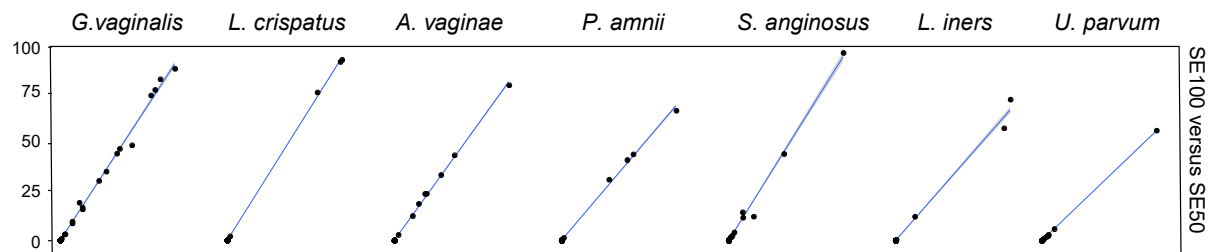**B**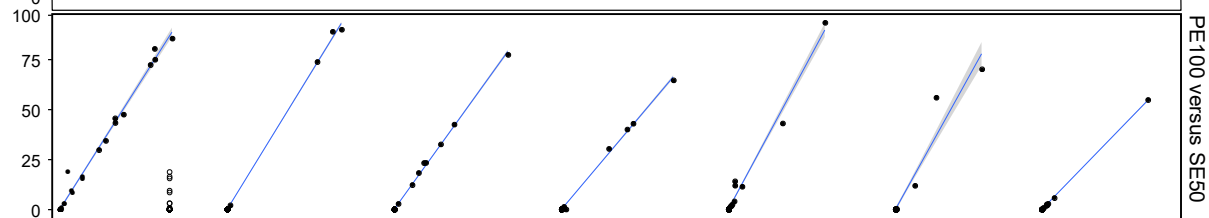**C**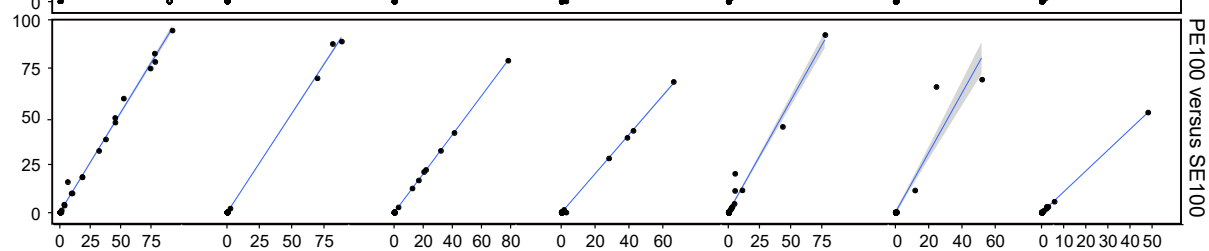

Supplement: Supplementary Figure S6 — Comparisons of species relative abundance between the longer and truncated reads. The taxonomic profile from PE100, pseudo-SE100, and pseudo-SE50 are compared by truncating the paired reads of 45 postmenopausal samples in the second cohort. The relative abundance of the top 7 species in the samples are shown. X-axis in the three panels denote using the pseudo-SE50 (A), pseudo-SE50 (B), and pseudo-SE100 (C) mode, respectively. Y-axis in the three panels denote using the pseudo-SE100 (A), PE100 (B), and PE100 (C) mode, respectively. A blank spot denotes a sample. The fitting blue line is robust linear model regress one mode against another mode. [file mmc6.pdf]

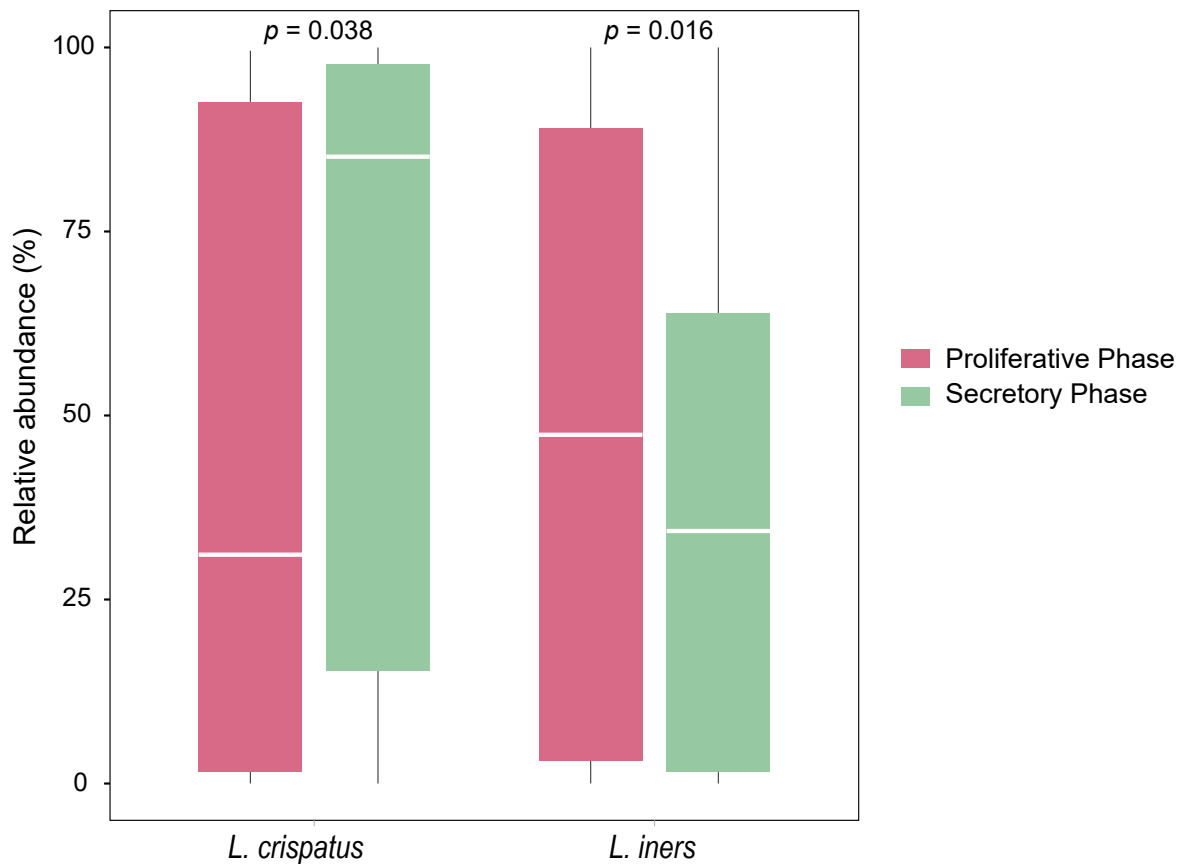

Supplement: Supplementary Figure S7 — Shift of L. crispatus and L. iners showing significant difference during menstrual cycle in the initial study cohort. The length of menstrual cycle of each woman is normalized to 28 days. The proliferative phase is from the end of the period to day 14, and the secretory phase is from the 15th to 28th day of a menstrual cycle. P value is calculated using Wilcoxon ranked sum test. The boxes denote the IQR between the first and third quartiles (25th and 75th percentiles, respectively), and the line inside the boxes denotes the median. The whiskers denote the lowest and highest values within 1.5 times the IQR from the first and third quartiles, respectively. IQR, interquartile range. [file mmc7.pdf]

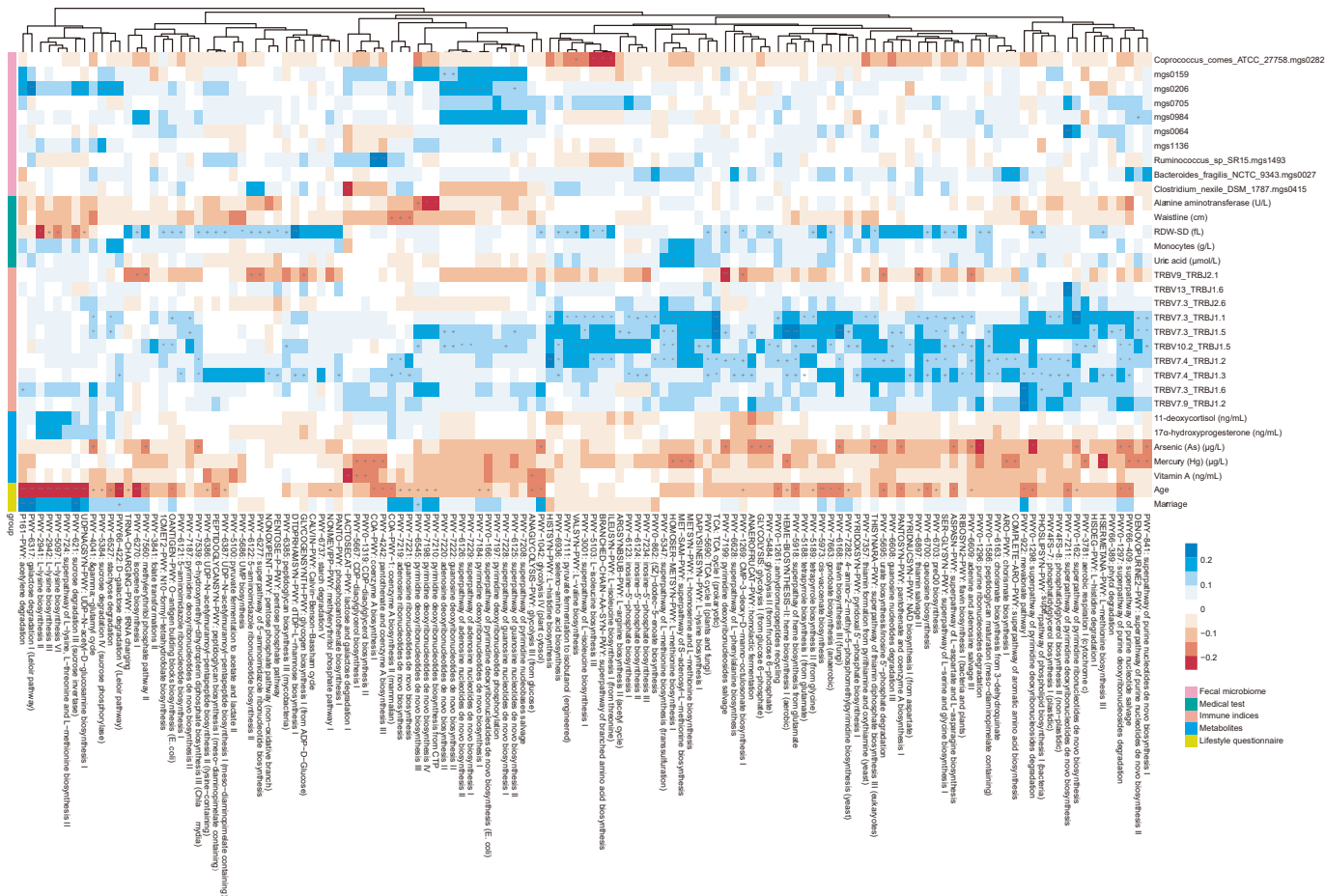

Supplement: Supplementary Figure S8 — Association between functional pathways of the vagino-cervical microbiome and multi-omics in the initial study cohort. Spearman’s correlation between pathway profiles according to HUMAnN2 and the numerical data from multi-omics. Only those associated significantly with at least one pathway are shown (“+” denotes Q value < 0.1, “*” denotes Q value < 0.05, “**” denotes Q value < 0.01). [file mmc8.pdf]

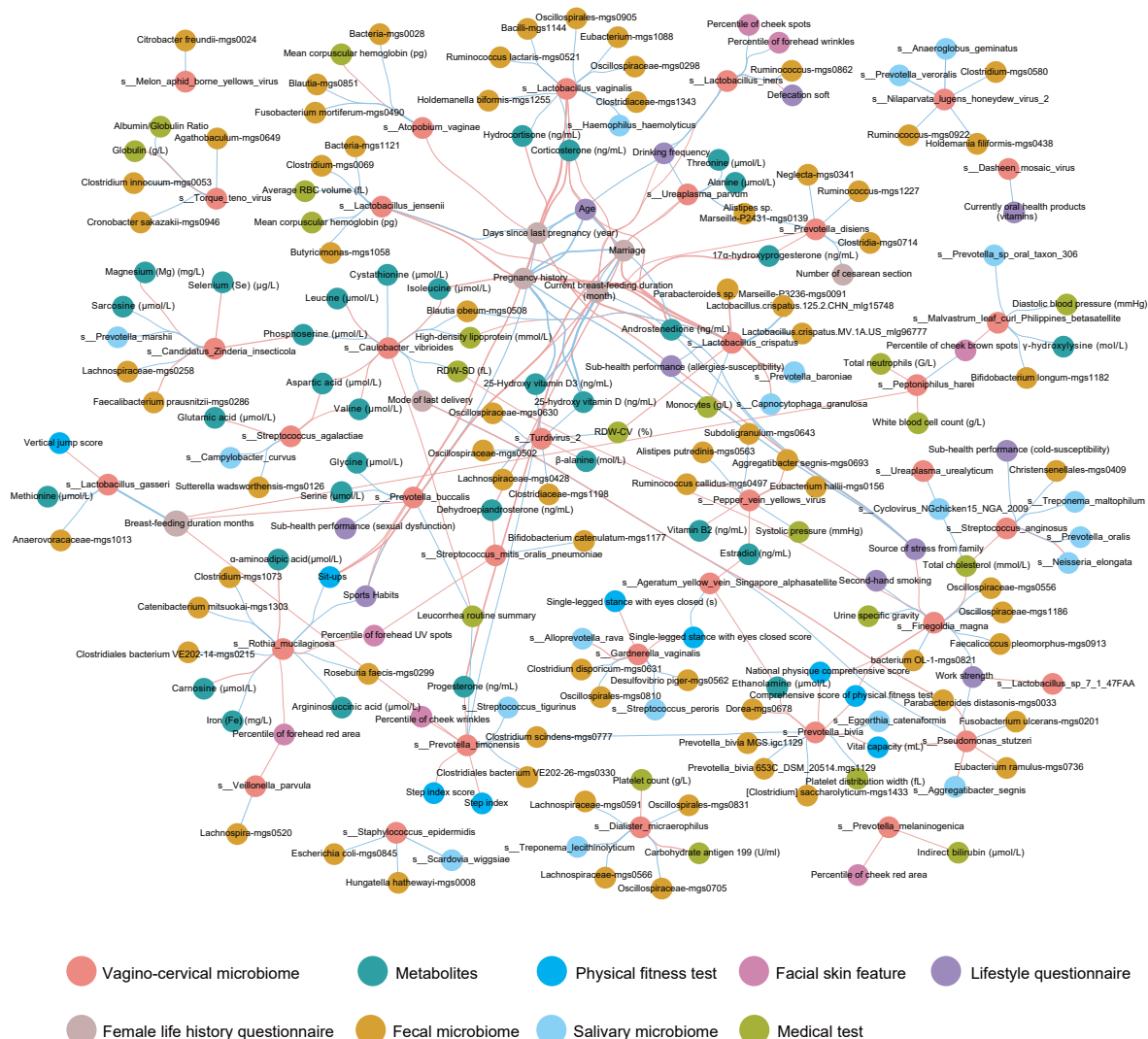

Supplement: Supplementary Figure S9 — Wisdom of the crowds for the association network between vagino-cervical microbial species and other multi-omic data in the second cohort. Results from generalized linear model with penalty (cv.glmnet), random forest (RFCV), and Spearman’s correlation are integrated and then visualized in Cytoscape. Red lines for negative associations; cyan lines for positive associations. [file mmc9.pdf]

A

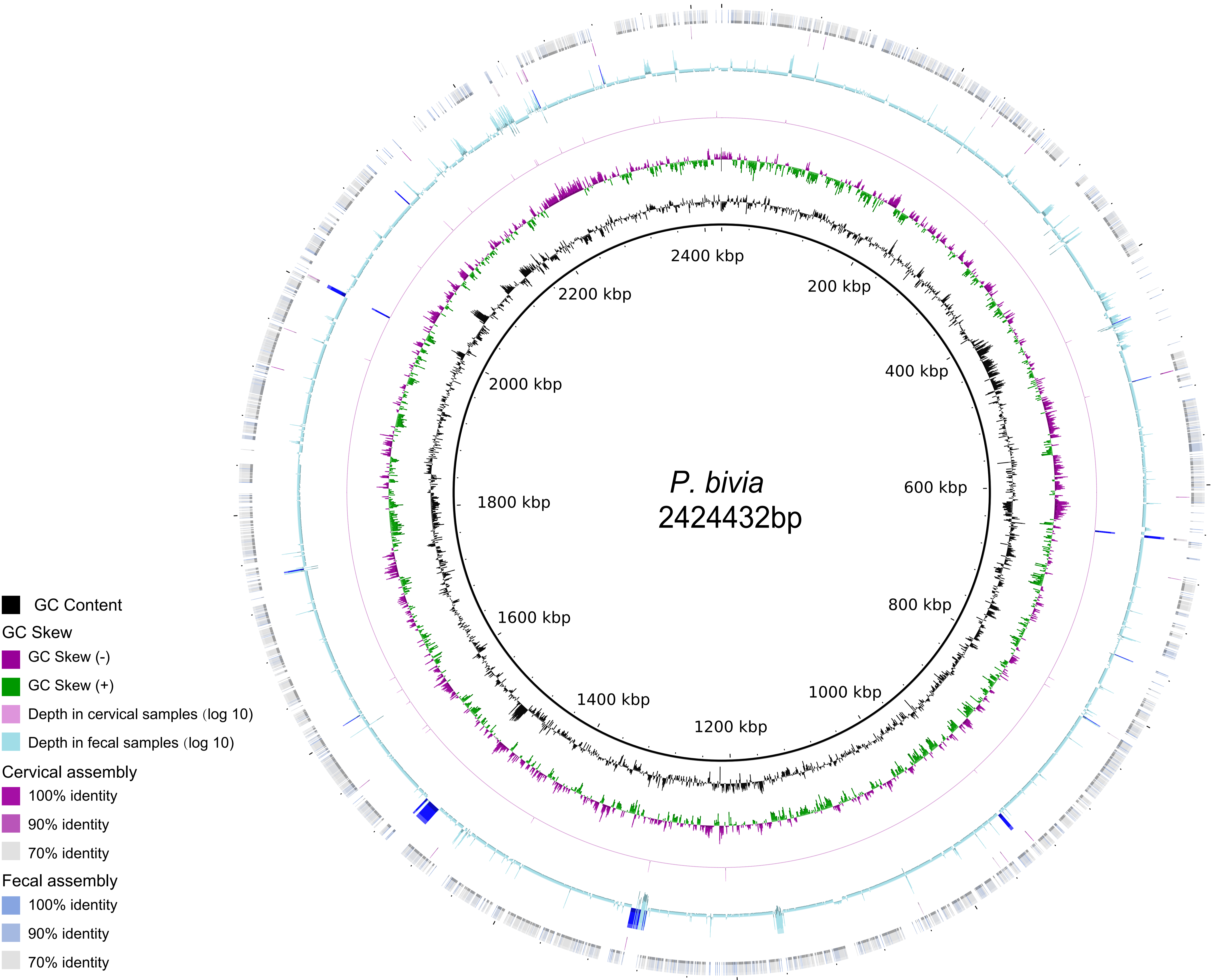

B

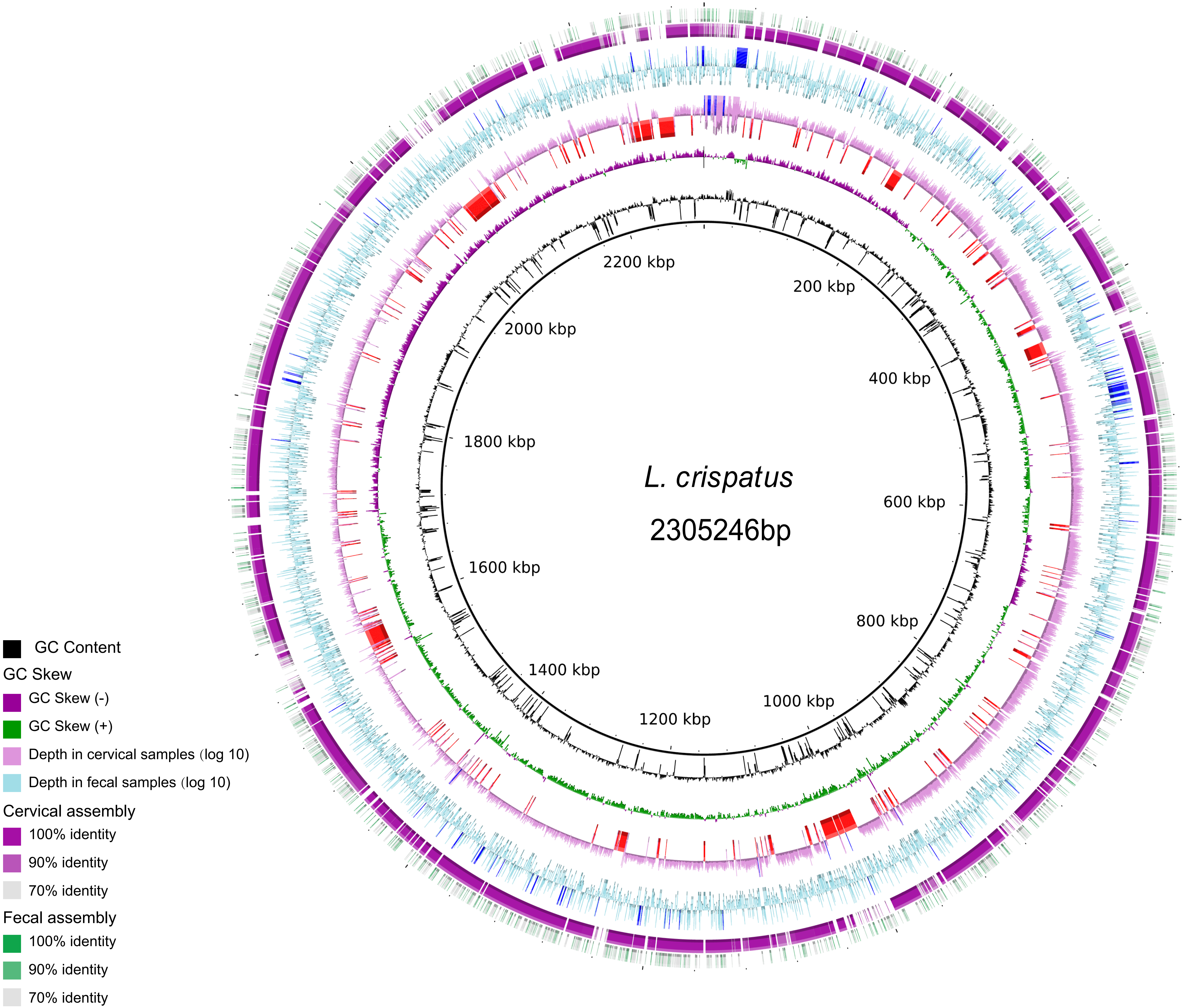

Supplement: Supplementary Figure S10 — Circular genome map of P. bivia (A) and L. crispatus (B) assembled from two separate individuals, including their fecal and vaginal samples. The inner to outer circles show (i) the GC content (black); (ii) the GC skew (+/−): values > 0 (green) and values < 0 (purple); (iii) the sequencing depth with log10-transformed of vaginal sample: values > 0 (pink) and values < 0 (red); (v) the sequencing depth with log10-transformed of fecal sample: values > 0 (light blue) and values < 0 (blue); (vi) assembled contigs from vaginal metagenomic shotgun data; (vii) assembled contigs from fecal metagenomic shotgun data. [file mmc10.pdf]
