# Supplementary material for: Life History Recorded in the Vagino-cervical Microbiome Along with Multi-omes
Source: Genomics Proteomics Bioinformatics. 2021 Jun 9;20(2):304–21. doi: 10.1016/j.gpb.2021.01.005 (PMC9684086; doi:10.1016/j.gpb.2021.01.005)
Supplement: Supplementary Figure S2 — The ratio of volunteers with breakdown experience in pregnancy history in the initial cohort. The different colors represent for the volunteers who experienced 1–6 times of having the experience of pregnancy, delivery, caesarean section, and abortion. N/A means lack of this data. [file mmc2.pdf]

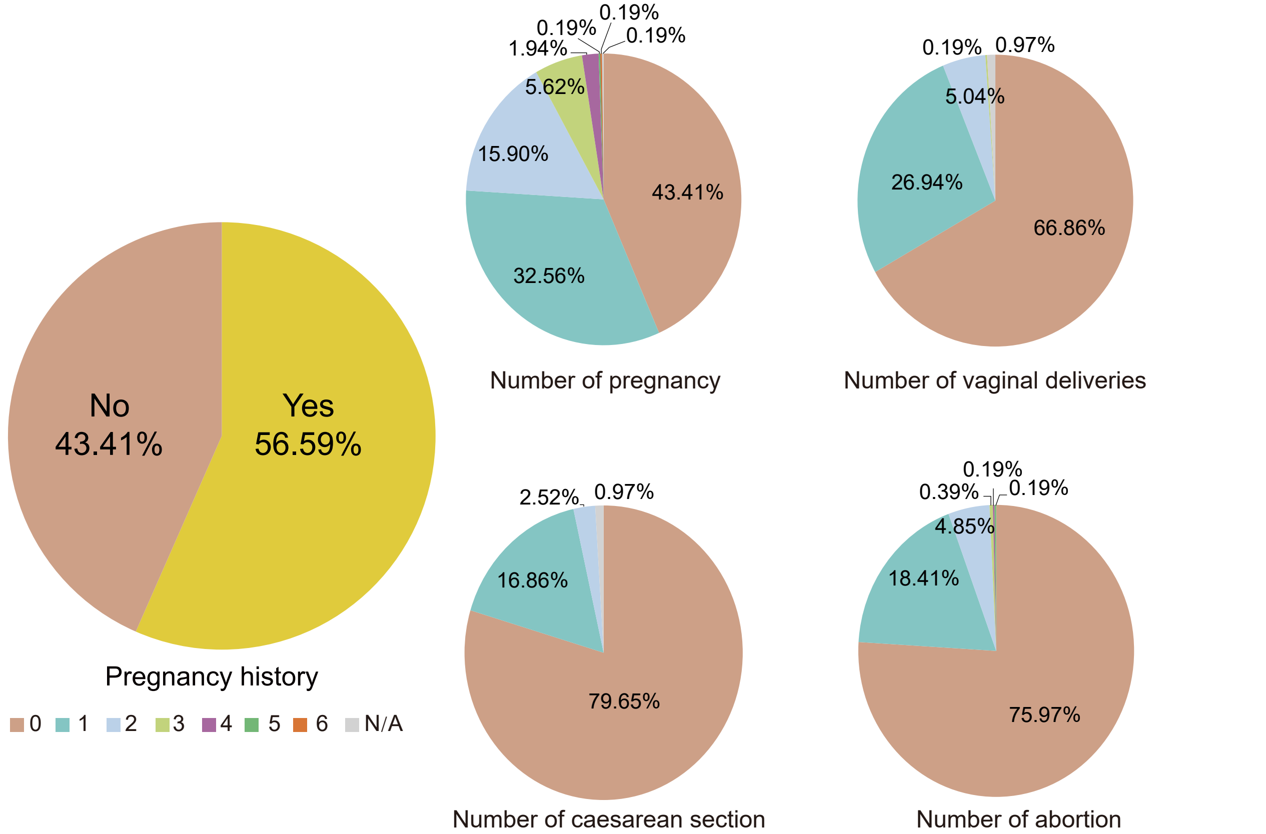

No
43.41%
Yes
56.59%
Pregnancy history
0
1
2
3
4
5
6
N/A
43.41%
32.56%
15.90%
5.62%
1.94%
0.19%
0.19%
0.19%
Number of pregnancy
66.86%
26.94%
5.04%
0.19%
0.97%
Number of vaginal deliveries
79.65%
16.86%
2.52%
0.97%
Number of caesarean section
75.97%
18.41%
4.85%
0.39%
0.19%
0.19%
Number of abortion
